# A comparative analysis of remdesivir and other repurposed antivirals against SARS‐CoV‐2

**DOI:** 10.15252/emmm.202013105

**Published:** 2020-11-03

**Authors:** Alexander Simonis, Sebastian J Theobald, Gerd Fätkenheuer, Jan Rybniker, Jakob J Malin

**Affiliations:** ^1^ Department I of Internal Medicine Division of Infectious Diseases University of Cologne Cologne Germany; ^2^ Faculty of Medicine Center for Molecular Medicine Cologne (CMMC) University of Cologne Cologne Germany; ^3^ German Center for Infection Research (DZIF) Partner Site Bonn‐Cologne Cologne Germany

**Keywords:** antivirals, COVID‐19, remdesivir, SARS‐CoV‐2, Microbiology, Virology & Host Pathogen Interaction, Pharmacology & Drug Discovery

## Abstract

The ongoing SARS‐CoV‐2 pandemic stresses the need for effective antiviral drugs that can quickly be applied in order to reduce morbidity, mortality, and ideally viral transmission. By repurposing of broadly active antiviral drugs and compounds that are known to inhibit viral replication of related viruses, several advances could be made in the development of treatment strategies against COVID‐19. The nucleoside analog remdesivir, which is known for its potent *in vitro* activity against Ebolavirus and other RNA viruses, was recently shown to reduce the time to recovery in patients with severe COVID‐19. It is to date the only approved antiviral for treating COVID‐19. Here, we provide a mechanism and evidence‐based comparative review of remdesivir and other repurposed drugs with proven *in vitro* activity against SARS‐CoV‐2.

## Graphical abstract

This comprehensive review discusses preclinical and clinical outcomes of remdesivir and other repurposed antiviral drugs against SARS‐CoV‐2.

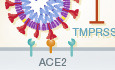

**Glossary**
Antiviral drugs: Drugs that directly interfere with the ability of a virus to replicate in vivo or in cell‐based models. Most antiviral drugs interfere with the host cell‐dependent life cycle of the virus. Thus, mode of action of most antivirals is the inhibition of the viral entry into the host cell, blockage of viral proteases, or inhibition of viral RNA replicase.
Bioavailability: Used to describe the fraction of a drug or its active metabolite that reaches the systemic circulation and organ tissue after administration.
Cell‐based assay: The term cell‐based assay is commonly used to refer to any assay, where living cells are used as model to study physiologic or pathophysiologic processes under various conditions (e.g., exposure to an antiviral agent). Due to their cost efficiency and high standardization/reproducibility, cell‐based assays are essential tools in preclinical drug discovery.
COVID‐19 (coronavirus disease 2019): The infectious disease caused by SARS‐CoV‐2 in humans.
Coronaviruses (CoV): Coronaviruses are a group of RNA viruses that cause diseases in mammals and birds. Coronavirus‐associated diseases in humans include severe acute respiratory syndrome (SARS), Middle East respiratory syndrome (MERS), and coronavirus disease 2019 (COVID‐19). In addition, there are endemic human CoVs that cause mild respiratory infections.
Drug repositioning or repurposing: A term that describes a drug discovery strategy based on the identification of new therapeutic approaches by using already known substances that may be at a preclinical or clinical development stage. This strategy offers a time‐ and cost‐saving method to develop therapeutics against newly emerged or neglected diseases.
Half‐maximal effective concentration (EC50): The concentration of a substance which is required to obtain 50% of its maximal effect. It is used to determine potency of a drug. For some analyses (for example antibacterial activity), the 50% inhibitory concentration (IC_50_) is used in analogy. Besides the half‐maximal concentration, the 90% maximal effective concentration (EC_90_) can be determined.
MERS‐CoV: Middle East respiratory syndrome‐related coronavirus causes the Middle East respiratory syndrome (MERS) in humans which is associated with severe respiratory symptoms and high mortality. The first confirmed case of MERS was reported in 2012.
Nucleoside/nucleotide analogs: Nucleosides are endogenous compounds composed of a nucleobase and a five‐carbon sugar (ribose or 2'‐deoxyribose), while nucleotides contain one more phosphate group. Nucleosides/nucleotides are essential for the synthesis of DNA and RNA but are also involved in other cellular processes like signaling and metabolism. Nucleoside/nucleotide analogs are synthetic, chemically modified nucleosides/nucleotides that are able to mimic their physiological counterparts. Assembly of nucleoside/nucleotide analogs into the RNA/DNA leads to premature termination of the strand synthesis and inhibition of, e.g., viral replication.
Pseudovirions/pseudotyped particles: Pseudovirions are synthetic viral particles with modified genomes and/or envelope proteins in order to facilitate specific investigations. The particles usually lack genes essential for pathogenicity and cannot replicate. This is an advantage for experiments on otherwise highly pathogenic viruses like SARS‐CoV‐2. Pseudotyping is the combination of viral particles with foreign viral envelope proteins. Pseudotyping can be used to study the function of viral envelope proteins and mechanisms of viral entry.
SARS‐CoV: The severe acute respiratory syndrome (SARS) coronavirus was first described in 2003. It causes a respiratory disease that accompanies a high rate of complications and mortality. After the epidemic outbreak in Asia in 2002‐2003, sporadic cases have been observed in several countries until 2004.
SARS‐CoV‐2: Severe acute respiratory syndrome coronavirus 2, initially described as 2019‐nCoV, causes respiratory infections that can progress to viral pneumonia in COVID‐19. It emerged in December 2019 in Wuhan, China, and rapidly developed to a pandemic which is still ongoing.

## Introduction

Coronaviruses (CoV) are known to cause respiratory tract infections in humans and animals. Since the emergence and subsequent characterization of the severe acute respiratory syndrome coronavirus (SARS‐CoV) in 2002 (Drosten *et al*, 2003; Ksiazek *et al*, 2003; Peiris *et al*, 2003) and Middle East respiratory syndrome coronavirus (MERS‐CoV) in 2012 (Corman *et al*, 2012), coronaviruses have increasingly been recognized as potential source of epidemic diseases. Both pathogens seem to cause zoonotic infections that originate from viral reservoirs in bats (Guan *et al*, 2003; Li *et al*, 2005; Mohd *et al*, 2016). In 2020, a novel coronavirus (SARS‐CoV‐2) emerged in China (Zhu *et al*, 2020) and spread globally in a very short period of time. The rapid geographical extension of SARS‐CoV‐2 in comparison to previous outbreaks with SARS‐CoV and MERS‐CoV may be caused by an increased infectivity of the pathogen (Sigrist *et al*, 2020; Wrapp *et al*, 2020). As of September 22, the ongoing coronavirus disease 2019 (COVID‐19) pandemic caused over 31 million detected SARS‐CoV‐2 infections and more than 950,000 deaths (Johns Hopkins University, 2020). The dramatic global implications of this pandemic stressed the urgent need for therapeutic agents that can quickly be applied in the clinic without a long‐lasting preclinical development phase. Several therapeutic strategies were therefore investigated by repurposing of known antimicrobial or immunomodulatory substances that might be beneficial for patients with COVID‐19. These agents can roughly be divided into compounds with a direct antiviral effect that impairs viral replication and host‐directed drugs that may support recovery from COVID‐19 by attenuating an excessive host immune response. In this article, we focus on repurposed drugs against COVID‐19 with proven antiviral effects against SARS‐CoV‐2 in cell‐based studies.

The most advanced developed antiviral of this type is the nucleoside analog remdesivir that was previously unsuccessfully tested against Ebolavirus disease in clinical trials (Mulangu *et al*, 2019). Based on recent clinical and preclinical data on its efficacy against COVID‐19, remdesivir received emergency use authorizations (EMA) in the United States and Japan and was recently approved by the European Medicines Agency (EMA) for the treatment of adult patients with severe COVID‐19 that require supplemental oxygen. Although approval of this drug is a very encouraging signal, its clinical efficacy seems to be relatively modest based on available evidence (Beigel *et al*, 2020; Goldman *et al*, 2020; Grein *et al*, 2020; Wang *et al*, 2020c). We will review preclinical and clinical outcomes of repurposed antivirals and their molecular mechanism of action (MOA) to provide a comparative analysis of remdesivir with the ultimate aim to support a rational appraisal of its efficacy.

### SARS‐CoV‐2 life cycle

The viral life cycle of SARS‐CoV‐2 provides several attractive molecular targets for viral inhibition that can be exploited by repurposed antiviral drugs. Like all *Coronaviriade*, this β‐coronavirus, is an enveloped, positive‐sense, single‐stranded RNA virus. It is composed of a core structure where the viral RNA is encapsulated by the nucleocapsid (N) protein and the envelope, a lipid bilayer in which the spike (S), membrane (M), and envelope (E) protein are anchored (de Haan & Rottier, 2005). Upon viral transmission, mostly via droplet transmission, the life cycle of SARS‐CoV‐2 is initiated by the attachment of the virion to the host cell by the spike glycoprotein (S‐protein) and its receptor. Several studies could show that entry, as shown for SARS‐CoV before, depends on binding of the receptor‐binding domain (RBD) (subunit S1) of the S‐protein to the human angiotensin converting enzyme receptor 2 (ACE2; Hoffmann *et al*, 2020a; Walls *et al*, 2020). Notably, the RBD of SARS‐CoV‐2 shows a 10‐ to 20‐fold higher affinity to ACE2 than SARS‐CoV, which may explain its increased transmissibility (Wrapp *et al*, 2020). Furthermore, single‐cell RNA‐sequencing data revealed a high expression level of the ACE2 receptor in human nasal epithelial cells, which may also enhance the efficiency of SARS‐CoV‐2 transmission (Sungnak *et al*, 2020). After initial binding of the S1 subunit to ACE2, entry into the host cell required proteolytic cleavage of the S‐protein at the S1/S2 and S2’ site, which leads to fusion of the viral and cellular membrane mediated by the S2 subunit. Proteolytic cleavage of the S‐protein is induced by the membranous serine protease TMPRSS2 of the host cell (Hoffmann *et al*, 2020a). Interestingly, a new furin cleavage site at the S1/S2 boundary could be found in SARS‐CoV‐2. The exact role of this site in pathogenesis is controversially discussed (Walls *et al*, 2020; Xia *et al*, 2020a). Cleavage of S‐protein exposes the S2 subunit which contains an internal fusion peptide and two hydrophobic (heptad) repeat regions (HR1 and HR2). HR1 and HR2 self‐assemble into a stable helical bundle that brings viral and cellular membranes in close proximity for fusion. Several bundles can form a fusion pore and finally release the viral genome into the cytoplasm (Bosch *et al*, 2003; Xia *et al*, 2020b). Moreover, several studies could show that virus entry is not only ensued by direct fusion with the plasma membrane, but rather by endosomal/lysosomal uptake and intra‐lysosomal activation of the spike protein by cathepsin L followed by membrane fusion and intracellular release of genomic RNA (Wang *et al*, 2008; Burkard *et al*, 2014; Ou *et al*, 2020).

**Figure 1:**
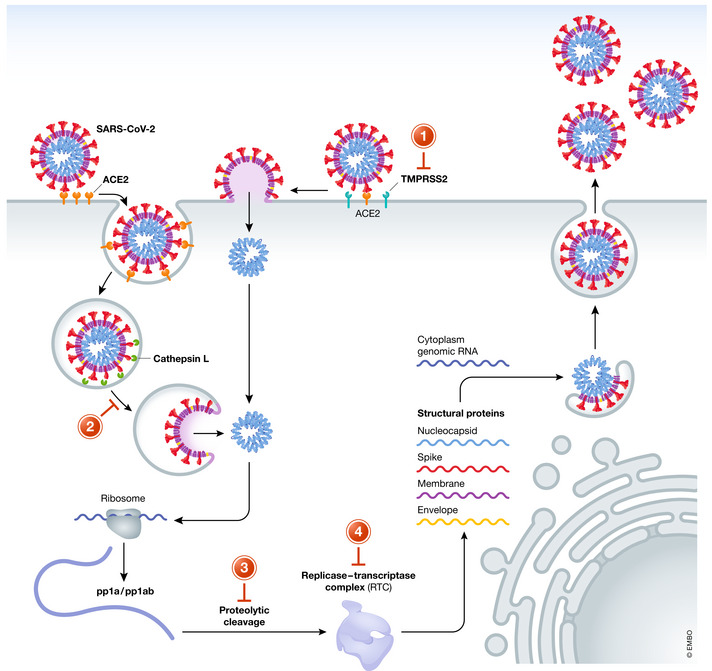
Life cycle of SARS‐CoV‐2 and antiviral drug targets Attachment of SARS‐CoV‐2 to its host cell is mediated by binding of the viral spike protein to the ACE2 receptor. After proteolytic cleavage of the S1 domain by the membrane‐anchored serine protease TMPRSS2, fusion of the viral and host cell membrane is initiated by the exposed S2 subunit. Alternatively, SARS‐CoV‐2 can invade the host cell upon endosomal uptake and activation of the spike protein by cathepsin L. Released viral RNA is translated by ribosomes of the host cell. Polyproteins pp1a/pp1ab are cleaved mainly by the viral main protease (3C‐like proteinase). Released non‐structural proteins form the replicase–transcriptase complex, which initiates the viral RNA synthesis machinery. Viral structure proteins and genomic RNA form new particles, which are released by exocytosis. The replication cycle of SARS‐CoV‐2 can be inhibited at various stadiums: viral entry (1‐2); protease inhibition (3), and RNA replication (4).

After release of viral RNA into the cytosol viral replication is initiated by the translation of the replicase gene encoded by two large ORFs (rep1a and rep1b), which express the two polyproteins pp1a and pp1ab. The polyproteins contain several non‐structural proteins (nsp) (pp1a = nsp 1–11; pp1ab = 1–16) also including a RNA‐dependent RNA polymerase (RdRp) domain (nsp12) and proteases that cleave the polyproteins (initiated by the enzyme's own autolytic cleavage from pp1a and pp1ab) (Anand *et al*, 2003; Pertusati *et al*, 2012). Most of the nsp forms the replicase–transcriptase complex (RTC): The RTC replicates the genomic RNA and sub‐genomic RNA, which encodes the structural proteins and other accessory proteins. While the nucleocapsid (N) protein remains in the cytosol and forms complexes with the genomic RNA, the viral structure proteins M, E, and S are translated, inserted into the membrane of the rough endoplasmatic reticulum (ER) and subsequently transported to the ER‐to‐Golgi intermediate compartment (ERGIC) (Fehr & Perlman, 2015). Here, the genomic RNA–nucleocapsid complexes get enveloped by the virion precursors, are transported to the cell surface in vesicles, and are released by exocytosis. An overview of the life cycle of SARS‐CoV‐2 including targets that might be exploited for inhibition of viral replication is illustrated in Figure 1. Based on its MOA, repurposed drugs with anti‐SARS‐CoV‐2 activity can be divided into substances that prevent viral entry into host cells (1–2) and inhibit viral proteases (3) and inhibitors of viral replicase (4). Other compounds elicit multiple effects, or its specific MOA in SARS‐CoV‐2 is unknown.

## Prevention of viral entry into the host cell

Viral entry is initiated by the S2 subunit, which requires prior S‐protein priming by proteolytic cleavage of the S1 subunit. As shown for other coronaviruses, viral entry in cell lines depends on the serine protease TMPRSS2 and the endosomal cysteine proteases cathepsin B and L (Kawase *et al*, 2012; Hoffmann *et al*, 2020a). However, several studies indicate that cell entry is driven preferentially via the cell surface or early endosomes by TMPRSS2 and that proteolytic cleavage of the S‐protein by TMPRSS2 is crucial for infection of the host (Shirato *et al*, 2018; Iwata‐Yoshikawa *et al*, 2019). Thus, inhibition of the TMPRSS2 and/or cathepsin B and L seems a promising target to prevent virus entry.

### Camostat/Nafamostat

TMPRSS2 is a cell membrane‐anchored serine protease and belongs to the family of type II transmembrane serine proteases. These proteases share a common catalytic mechanism involving a triad of three amino acids, serine, aspartate, and histidine present in highly conserved sequence motifs (Antalis *et al*, 2011). Serine proteases underlie a strict regulation by endogenous inhibitors (e.g., α1/α2‐antitrypsin, and antithrombin III) and need a prior activation leading to hemostasis under physiological conditions. Thus, imbalance can cause several pathophysiological processes like thrombosis (Rau *et al*, 2007). However, the exact physiological functions of TMPRSS2 are still unknown. Synthetic protease inhibitors like camostat mesilate or nafamostat mesilate have been clinically tested in patients with acute or chronic pancreatitis, which is pathophysiologically related to an inappropriate activation of digestive enzymes inside the pancreas, including the serine protease trypsin (Chang *et al*, 2009; Ramsey *et al*, 2019). Due to their capability to inhibit TMPRSS2, serine protease inhibitors have been tested for their antiviral effects on SARS‐CoV‐2 and other coronaviruses. Camostat partially blocked the entry of vesicular stomatitis virus (VSV) pseudotyped particles harboring the SARS‐CoV‐2 spike protein (pseudovirions) into the human epithelial colorectal adenocarcinoma cell line Caco‐2, Vero‐TMPRSS2^+^ cells, and human airway epithelial (HAE). A complete inhibition of viral entry could only be reached when camostat was used in combination with E‐64d, an inhibitor of cathepsin B/L, suggesting that SARS‐CoV‐2 can exploit both pathways for entry into the host cell (Hoffmann *et al*, 2020a). However, TMPRSS2 is essential for viral transmission and pathogenesis while CatB/L activity is dispensable so that inhibition of TMPRSS2 displays a rational antiviral strategy (Iwata‐Yoshikawa *et al*, 2019).

Wang *et al* demonstrated inhibition of SARS‐CoV‐2 by nafamostat with a 50% effective inhibitory concentration (EC_50_) of 22.50 μM in Vero E6 cells (Wang *et al*, 2020a). A comparative assessment of the serine protease inhibitors gabexate mesilate, camostat mesilate, and nafamostat mesilate and their ability to inhibit viral entry was done by Hoffmann *et al* Efficiency of entry inhibition was determined 16 h post‐inoculation by using Calu‐3 cells infected with SARS‐CoV‐2‐pseudovirions. Nafamostat demonstrated an almost 15‐fold higher efficiency (EC_50_ 5 nM) compared with camostat (87 nM), both superior to gabexate (EC_50_ 1.2 M). Nafamostat also showed to inhibit SARS‐CoV‐2 infection of lung‐derived human Calu‐3 cells *in vitro* even at a low dose of 100 nM (Hoffmann *et al*, 2020b).

Although antiviral efficacy of TMPRSS2 inhibitors seems to be inferior to other strategies (table 1), entry inhibitors may be developed that are beneficial in COVID‐19 when given alone or in combination with other antivirals. Three randomized controlled trials (RCT) are currently listed that evaluate nafamostat in patients with COVID‐19 (NCT04418128, NCT04352400, NCT04473053), but currently no clinical data can be reported.

**Table 1:** *In vitro* efficacy and drug targets of repurposed investigational compounds with proven anti‐SARS‐CoV‐2 activity

Antiviral target	Investigational drug	Isolate	EC_50_ *in Vero E6 cells (µM)*			References
			CPE	RT–PCR	VY	
Viral entry	Nafamostat	Wuhan/WIV04/2019	ND	22.50	ND	Wang *et al* (2020)
	Umifenovir (Arbidol)	Wuhan/WIV04/2019	ND	4.11	ND	Wang *et al* (2020b)
		France/IDF0571/2020	ND	3.54	ND	Pizzorno *et al* (2020)
Viral protease	Lopinavir	Hong Kong/VM20001061/2020	25^a^	26.10^a^	26.62^a^	Choy *et al* (2020)
		France/IDF0571/2020	ND	5.25	ND	Pizzorno *et al* (2020)
RNA synthesis	Favipiravir	Wuhan/WIV04/2019	ND	61.88	ND	Wang *et al* (2020)
		Hong Kong/VM20001061/2020	> 100	> 100	> 100	Choy *et al* (2020)
		France/IDF0571/2020	ND	> 100	ND	Pizzorno *et al* (2020)
	Penciclovir	Wuhan/WIV04/2019	ND	95.96	ND	Wang *et al* (2020)
	Remdesivir	Wuhan/WIV04/2019	ND	0.77	ND	Wang *et al* (2020)
		Australia/VIC01/2020	4.9	ND	ND	Ogando *et al* (2020)
		Hong Kong/VM20001061/2020	25^a^	26.9^a^	23.15^a^	Choy *et al* (2020)
		France/IDF0571/2020	ND	0.99	ND	Pizzorno *et al* (2020)
	Ribavirin	Wuhan/WIV04/2019	ND	109.50	ND	Wang *et al* (2020)
		Hong Kong/VM20001061/2020	500^a^	> 500	> 500	Choy *et al* (2020)
Miscellaneous	Berberine	France/IDF0571/2020	ND	10.58	ND	Pizzorno *et al* (2020)
	Chloroquine	Wuhan/WIV04/2019	ND	1.13	ND	Wang *et al* (2020)
		France/IDF0571/2020	ND	1.38	ND	Pizzorno *et al* (2020)
		Wuhan/WIV04/2019	ND	2.71‐7.36^b^	ND	Liu *et al* (2020)
		Wuhan/IVDC‐HB‐01/2019	ND	5.47	ND	Yao *et al* (2020)
	Hydroxychloroquine	Wuhan/WIV04/2019	ND	4.06‐12.96^b^	ND	Liu *et al* (2020)
		Wuhan/IVDC‐HB‐01/2019	ND	0.72	ND	Yao *et al* (2020)
		France/lDF0372/2020	ND	2.2‐4.4^c^	ND	Maisonnasse *et al* (2020)
	Cyclosporine A	France/IDF0571/2020	ND	3.05	ND	Pizzorno *et al* (2020)
	Emetine	Hong Kong/VM20001061/2020	1.56^a^	0.50^a^	0.46^a^	Choy *et al* (2020)
	Homoharringtonine	Hong Kong/VM20001061/2020	3.13^a^	2.14^a^	2.55^a^	Choy *et al* (2020)
	Nitazoxanide	Wuhan/WIV04/2019	ND	2.12	ND	Wang *et al* (2020)

EC_50_, 50% effective concentrations.

Assay types: CPE, cytopathologic effects; RT–PCR, real‐time polymerase chain reaction; VY, virus yield assay.

^a^ Calculation of EC_50_ based on viral loads fitted to log_10_ scale.

^b^ Tested in different MOI (0.01, 0.02, 0.8).

### Umifenovir

Umifenovir is a broad‐spectrum antiviral approved in Russia and China for the prophylaxis and treatment of human influenza A and B infections (Boriskin *et al*, 2008). Its antiviral mechanism of action is thought to be related to an impaired virus‐mediated membrane fusion that is essential for viral entry. Umifenovir seems to modify the physicochemical properties of the host cell membrane by influencing the negatively charged phospholipids (Villalaín, 2010). Furthermore, it has been shown that umifenovir interacts with hemagglutinin (HA) of the influenza virus by preventing the pH‐induced transition of HA into its functional state (Leneva *et al*, 2009). In a recent study, the efficacy of six currently available and licensed anti‐influenza drugs (umifenovir, baloxavir, laninamivir, oseltamivir, peramivir, and zanamivir) were tested against SARS‐CoV‐2 in Vero E6 cells. Among tested drugs, only umifenovir inhibited SARS‐CoV‐2 replication efficiently with an EC_50_ of 4.11 μM (Wang *et al*, 2020b). These results could be reproduced by another *in vitro* study with an EC_50_ of 3.5 μM (Pizzorno *et al*, 2020). Although umifenovir demonstrated anti‐SARS‐CoV‐2 activity *in vitro*, a therapeutic role of umifenovir in COVID‐19 is uncertain and results of qualitative clinical trials are lacking. Retrospective analyses currently indicate no significant impact on clinical outcomes (Huang *et al*, 2020).

## Blockage of viral proteases

A crucial step in SARS‐CoV‐2 replication is the proteolytic cleavage and release of functional polypeptides from the polyproteins pp1a/pp1ab by viral proteases. Subsequently, released non‐structural proteins form the replicase–transcriptase complex, which initiates the viral RNA synthesis machinery. Translated viral structure proteins and replicated genomic RNA originate new infectious virus particles, which are released from the infected host cell. In coronaviruses, the main protease (Mpro) also known as 3C‐like protease (3CL^pro^) cleaves the polyprotein at conserved sites between Leu‐Gln and Ser‐Ala‐Gly. This well‐characterized enzyme represents an ideal antiviral target as its function is critical for viral replication (Anand *et al*, 2003; Zhang *et al*, 2020b). Due to its intrinsic proteolytic activity and the absence of homologous enzymes in humans, toxicity of specific inhibitors is expected to be limited. Of known protease inhibitors that were repurposed for SARS‐CoV‐2, the combination of lopinavir and ritonavir has been in focus of interest as other protease inhibitors (e.g., darunavir) showed no *in vitro* activity at applicable concentrations (De Meyer *et al*, 2020).

### Lopinavir/ritonavir

Lopinavir/ritonavir is used as combination regimen in the treatment of infections with human immune deficiency virus 1 (HIV‐1). Both lopinavir and ritonavir are inhibitors of HIV‐1 protease, an enzyme that cleaves the HIV polyproteins Gag and Gag‐Pol by bond hydrolysis. Since ritonavir also acts as inhibitor of cytochrome P450‐3A4 (CYP3A4), an enzyme that normally metabolizes protease inhibitors, ritonavir is added to enhance the bioavailability of lopinavir (Sham *et al*, 1998). Lopinavir has been tested *in vitro* against SARS‐CoV, MERS‐CoV, and human coronavirus 229E (de Wilde *et al*, 2014). Here, the mean EC_50_ of lopinavir ranged from 6.6 µM (± 1.1) µM (HCoV‐229E) and 8.0 µM (± 1.5) MERS‐CoV to 17.1 µM (± 1.0) (SARS‐CoV). Recent analysis demonstrated that lopinavir is also active against SARS‐CoV‐2 with an EC_50_ of 5.25–26.1 µM (Choy *et al*, 2020; Pizzorno *et al*, 2020) while ritonavir alone was not effective (Choy *et al*, 2020). *In vivo* efficacy of lopinavir/ritonavir has been assessed in mice and common marmosets for MERS‐CoV with ambiguous results: In a study published in 2015, lopinavir/ritonavir‐treated marmosets had improved clinical findings and reduced viral loads associated with a better outcome. Animals were treated with 2 mg/kg/day of lopinavir plus 3 mg/kg/day of ritonavir given orally once daily at 6, 30, and 54 h post‐infection (Chan *et al*, 2015). However, treatment of infected mice with lopinavir/ritonavir (160/40 mg + interferon beta) improved pulmonary function but did not reduce virus replication or occurrence of severe lung damage (Sheahan *et al*, 2020). Clinical effects in patients with severe COVID‐19 were evaluated in a randomized controlled clinical trial including 199 patients. Patients were randomized in a 1:1 ratio to receive either lopinavir/ritonavir (standard dose of 400/100 mg) for 14 days or the standard care. The primary end point of the study was clinical improvement or discharge from the hospital. Unfortunately, treatment did not improve clinical symptoms and mortality, or decreased viral loads in pharyngeal swabs (Cao *et al*, 2020). The disappointing clinical results might be related to sub‐therapeutic levels for inhibition of SARS‐COV‐2 because application of 400/100 mg of lopinavir/ritonavir twice daily was shown to yield median serum concentrations of 7.2 mg/l (11.5 µM) in patients with HIV (van der Lugt *et al*, 2009), which is significantly lower than the observed EC_50_ in the *in vitro* studies. However, summarizing the relatively low efficacy against SARS‐CoV‐2 *in vitro* in comparison with other repurposed drugs and available *in vivo* data it is unlikely that lopinavir/ritonavir will play a significant therapeutic role in COVID‐19. Besides lopinavir and ritonavir, other protease inhibitors with activity against SARS‐CoV and MERS‐CoV were identified that might be repurposed to target SARS‐CoV‐2 (Anand *et al*, 2003; He *et al*, 2020a).

## Inhibition of viral RNA replicase

Once functional, non‐structural proteins are released by proteolytic cleavage of the polyproteins, the replicase–transcriptase complex, which catalyzes the synthesis of the viral RNA, can be formed. Synthesis is initiated by binding of the RdRp at or near the 3' end of the RNA strand. Subsequently, the complementary RNA strand is generated in the elongation phase by repetitive nucleotidyl transfer reactions. Several drugs are able to interfere with the RNA synthesis machinery. Mainly, nucleoside/nucleotide analogs have been repurposed and tested against SARS‐CoV‐2. These drugs disrupt viral replication by competing with endogenous nucleosides during the elongation phase. After their insertion nucleoside analogs cause a chain termination followed by an abrogation of RNA synthesis, which is crucial to produce new viral particles.

### Remdesivir

Remdesivir (GS‐5734) is a prodrug of a monophosphoramidate nucleoside that is designed to easily pass the cell membrane and efficiently deliver its active metabolite (Jordheim *et al*, 2013). Upon entering the target cells, remdesivir monophosphate (RDV‐MP) is rapidly converted into its active triphosphate form due to its ability to bypass an inefficient and rate‐limiting first phosphorylation step (Murakami *et al*, 2008). In RNA viruses, the metabolically active remdesivir triphosphate (RDV‐TP) acts as substrate for the viral replicase (RdRp) where it competes with endogenous adenosine‐triphosphate (ATP) for incorporation in elongating RNA strands. After its incorporation, RDV‐TP causes a synthesis arrest by inducing delayed chain termination as demonstrated for Ebola virus (EBOV) (Tchesnokov *et al*, 2019), MERS‐CoV (Gordon *et al*, 2020a), SARS‐CoV, and SARS‐CoV‐2 (Gordon *et al*, 2020b). In SARS‐CoV‐2, incorporation of RDV‐TP causes termination of RNA synthesis after three additional nucleoside/nucleotide positions downstream (Gordon *et al*, 2020b). Although related analogs of RDV have been under investigation and pharmacological modification for many years (Cho *et al*, 2012; Seley‐Radtke & Yates, 2018; Yates & Seley‐Radtke, 2019), the current molecule as a candidate for the treatment of viral diseases was first described in 2016 based on preclinical data from cell‐based assays and a macaque model of fatal EVD (Warren *et al*, 2016).

In fact, RDV has a very broad antiviral activity spectrum among RNA viruses. Along with efficacy against EBOV and Marburg virus that belong to the *Filoviridae* family, it was shown that RDV effectively inhibits RNA viruses of the *Paramyxoviridae*, *Pneumoviridae,* and *Coronaviridae* families with EC_50_ in the sub‐micromolar range (Warren *et al*, 2016; Lo *et al*, 2017; Sheahan *et al*, 2017). Efficacy against SARS‐CoV and MERS‐CoV was mainly tested in human airway cells (HAE or Calu‐3). Using RT–PCR or reporter gene‐based assays, RDV yielded EC_50_ of 0.025–0.12 µM (MERS‐CoV) and 0.069–0.07 µM (SARS‐CoV) (Sheahan *et al*, 2017; Agostini *et al*, 2018; Sheahan *et al*, 2020). In addition, RDV inhibits zoonotic and epidemic human CoVs (Brown *et al*, 2019). Inhibitory effects on SARS‐CoV‐2 were evaluated in the African green monkey kidney cell line (Vero E6) that supports entry and replication of SARS‐CoV‐2 by a high expression of ACE2 (Banerjee *et al*, 2020; Hoffmann *et al*, 2020a). A clinical virus isolate from Wuhan (WIV04/2019) RDV was inhibited with an EC_50_ of 0.77 µM in a RT–PCR‐based assay (Wang *et al*, 2020a). Another group assessed the reduction of cytopathology effects (CPE) by RDV using an Australian isolate (VIC01/2020). Here, the EC_50_ was significantly higher (4.9 µM) which might reflect methodological differences as this increased level was in the same order to a similarly tested SARS‐CoV isolate (Ogando *et al*, 2020). Recently, another preclinical evaluation was done using a SARS‐CoV‐2 isolate from Hong Kong (20001061/2020). The investigators found EC_50_ between 23.12 µM and 25 µM in different assay formats. However, these result cannot be readily compared with previous findings because a logarithmic fitted calculation model was used (Choy *et al*, 2020).

RDV demonstrated beneficial therapeutic effects in several animal models of CoV infections including mouse models of SARS‐ and MERS‐CoV infection and in MERS‐CoV‐infected non‐human primates (Sheahan *et al*, 2017; de Wit *et al*, 2020; Sheahan *et al*, 2020). Here, it also had prophylactic properties when 5 mg/kg was administered 24 h before inoculation of rhesus macaques with MERS‐CoV. Recently, RDV was evaluated in a macaque model of SARS‐CoV‐2 infection. Animals were treated 12 h post‐infection with 10 mg/kg (day 1) followed by 5 mg/kg daily (day 2‐6) which is an equivalent dose of that recommended for humans (Gilead_Sciences, 2020). In contrast to animals treated with placebo (*n* = 6), RDV diminished clinical signs of disease and reduced lung virus titers and tissue damage in all six animals treated (Williamson *et al*, 2020).

Clinically, RDV was evaluated in two randomized controlled clinical trials of which results have been published. The first trial conducted in China was unfortunately underpowered (n_RDV_ = 158; n_placebo_ = 79) due to insufficient recruitment of patients and therefore remained inconclusive. However, in a subgroup of patients that were treated with RDV within 10 days of symptom onset there was a numerical reduction of five days in time to clinical improvement that was not yet significant (hazard ratio 1.52 [95% CI: 0.95–2.43]) (Wang *et al*, 2020c). The adaptive COVID‐19 treatment trial was an international double‐blind RCT that included 1063 patients. An interim analysis was performed after completion of enrollment, with a total of 301 patients in follow‐up (before day 29) that was made public to make positive results quickly available. In this preliminary evaluation, treatment with RDV was associated with a significant reduction in time to recovery from median 15 to 11 days (recovery rate ratio 1.32 [95% CI: 1.12–1.55; *P* < 0.001]) which was most pronounced in patients that require supplemental oxygen (RRR 1.47 [95% CI: 1.17–1.84]). Mortality rates by 14 days were reduced in the treatment group (7.1% vs. 11.9%), which was not yet statistically significant (HR 0.7 [95% CI: 0.47–1.03, *P* = 0.06]) (Beigel *et al*, 2020). Based on these findings, RDV was given an Emergency Use Authorization (EUA) in the United States and Japan and was recently approved by the European Medicines Agency for the treatment of patients with COVID‐19 that require supplemental oxygen. A randomized open‐label study sponsored by Gilead Sciences (NCT04292899) compared efficacy of a 10‐day (*n* = 197) vs. 5‐day treatment course (*n* = 200) in patients with severe COVID‐19. Results from this analysis suggest similar effects of both regimens. However, an overall assessment of RDV efficacy is not possible based on these data as no control group was included in this trial. Another Gilead Sciences sponsored phase 3 randomized controlled trial evaluating RDV in moderate COVID‐19 (NCT04292730) found a significant better clinical status by day 11 (primary outcome) in patients treated with a 5‐day regimen of RDV compared with placebo (odds ratio 1.65 [95% CI: 1.09–2.48, *P* = 0.02]). However, the clinical significance of this finding remains unclear because a 10‐day course had no influence on this outcome and the effect was inconsistent with another evaluation on day 28 (Spinner *et al*, 2020). Of all results from clinical trials on RDV, only one publication reports on its impact on SARS‐CoV‐2 viral load. Wang *et al* found similar decreases in virus RNA of upper and lower respiratory tract specimen of patients treated with either RDV or placebo. This finding may be of limited significance as the study was generally underpowered and only a limited number of patients was eligible for this evaluation (67% had a PCR‐positive upper respiratory specimen at baseline and expectorated sputa were obtained from 43% of enrolled patients) (Wang *et al*, 2020c).

Safety data of RDV are available from 138 healthy volunteers (phase I) and more than 1,500 patients treated within phase III trials on COVID‐19 or compassionate use programs (FDA, 2020). In general, RDV was well tolerated and serious adverse events seem to be rare. RDV is known to interfere with several hepatic drug‐metabolizing enzymes like CYP2C8, CYP2D6, and CYP3A4 *in vitro*. In healthy individuals, RDV increased the risk of transient transaminase elevations. However, in randomized clinical trials similar elevations were observed in both RDV and placebo groups which might be explained by COVID‐19‐associated liver injury (Fan *et al*, 2020; Lei *et al*, 2020; Zhang *et al*, 2020a). A complete overview of safety information for RDV can be reviewed elsewhere (FDA, 2020).

### Favipiravir

Favipiravir (T‐705) is an oral pyrazine derivate that inhibits RdRp of several RNA viruses. For influenza, it was shown that the active triphosphate form functions as a nucleotide analog that competes with ATP and guanosine‐triphosphate (GTP) for incorporation into the nascent RNA strand, thereby causing chain termination (Sangawa *et al*, 2013). In addition to its action as competitive inhibitor of viral RdRp, favipiravir‐TP triggers accumulation of random point mutations that ultimately lead to lethal mutagenesis of the virus (Vanderlinden *et al*, 2016; preprint: Shannon *et al*, 2020). The drug has potent antiviral activity against influenza A and B *in vitro* and is currently approved in Japan for the treatment of influenza infections (Furuta *et al*, 2002; Furuta *et al*, 2013). Furthermore, it demonstrated a broad antiviral spectrum against other RNA viruses like paramyxoviruses, human metapneumovirus, respiratory syncytial virus, human parainfluenza virus and measles virus (Jochmans *et al*, 2016). However, cell‐based assays that evaluated efficacy against SARS‐CoV‐2 showed only low activity at a high micromolar range (Wang *et al*, 2020a) or no activity at the highest concentration tested (Choy *et al*, 2020; Pizzorno *et al*, 2020). Despite its poor *in vitro* efficacy, favipiravir was evaluated in an open‐label non‐randomized trial which compared time to viral clearance and radiological improvement after 14 days treatment with either lopinavir/ritonavir (400/100 mg twice daily; n = 45 historical controls) or favipiravir (1600 mg d1, 600 mg d2‐14, twice daily; *n* = 35) plus the standard of care (SOC) in hospitalized patients with COVID‐19. The investigators found a significant shorter median time to viral clearance (4 days, IQR: 2.5–9 vs. 11 days, IQR: 8–13; *P* < 0.001) and a higher rate of patients with improved chest imaging on day 14 (91.43% vs. 62.22%; *P* = 0.004) in the group treated with favipiravir (Cai *et al*, 2020). However, these data are difficult to interpret as there was no placebo control and all patients received additional treatments with interferon (IFN)‐α 1b. Taken together, no convincing evidence for favipiravir as antiviral agent against SARS‐CoV‐2 can be reported. Based on its poor *in vivo* efficacy, it seems unlikely that this drug will be assessed in another clinical trial.

### Ribavirin

Ribavirin is a guanosine analog with structural similarities to favipiravir. Like other nucleoside or nucleotide analogs, it abrogates viral RNA synthesis by incorporation into nascent RNA strands. However, additional processes may also contribute to its antiviral activity. For influenza, for example it was shown that ribavirin provides a mutagenic effect on the viral genome and decreases cellular GTP pools by interfering with cellular inosinmonophosphat‐dehydrogenase (Streeter *et al*, 1973; Wray *et al*, 1985). Ribavirin is an approved drug for the treatment of chronic infections with hepatitis‐C virus (HCV) in combination with other antiviral drugs. Like other RdRp inhibitors, ribavirin has a broad activity among RNA viruses, especially in those belonging to the flavivirus family (Crance *et al*, 2003). Although virtual molecular docking studies do suggest an interaction with to SARS‐CoV‐2 RdRp, its efficacy against SARS‐CoV‐2 is very limited (Choy *et al*, 2020; Wang *et al*, 2020a). This is not surprising as ribavirin also lacks activity against related coronaviruses (Cinatl *et al*, 2003). Therefore, ribavirin was not evaluated *in vivo*.

### Penciclovir

Pencivlovir is another guanosine analog that is an approved antiviral for topical treatment of herpes simplex virus infections or reactivations. It is closely related to acyclovir but has a very poor bioavailability. Its prodrug form, famciclovir, has an optimized bioavailability and is used as systemic treatment for herpes infections including herpes zoster. Virtual binding studies based on an nsp12 homology model suggest that pencivlovir binds to SARS‐CoV‐2 RdRp with an affinity even higher than that of RDV (preprint: Dey *et al*, 2020). Nevertheless, it demonstrated low efficacy against SARS‐CoV‐2 *in vitro* (EC_50_ 96 µM) (Wang *et al*, 2020a). Additional preclinical or clinical studies with penciclovir or its prodrug famciclovir as treatment for COVID‐19 have not been reported.

## Miscellaneous or unknown MOA

### Chloroquine/Hydroxychloroquine

Chloroquine (CQ) is a 9‐aminoquinoline that has been used as anti‐malaria drug for decades but its use steadily decreased because of emerging resistant *Plasmodium falciparum* (Wellems & Plowe, 2001). CQ and its derivate hydroxychloroquine (HCQ) however are still in clinical use to treat rheumatic diseases where it has beneficial immunomodulatory effects. Hydroxychloroquine demonstrated less toxicity in animal studies that tested high doses in mice, rats, and dogs (McChesney, 1983). In the past years, CQ/HCQ has gained attention for its potential use as therapeutic agent in the field of bacterial and viral infectious diseases because of its ability to inhibit several intracellular bacteria, viruses, and fungi (Savarino *et al*, 2004; Rolain *et al*, 2007).

The MOA of CQ/HCQ is not completely understood and varies among different pathogens to some extent. In general, non‐protonated forms of CQ/HCQ enter the cell and subsequently become protonated according to the Henderson‐Hasselbach law (Savarino *et al*, 2004). Consequently, CQ/HCQ accumulates in acidic organelles, such as endosomes, lysosomes, and Golgi vesicles. Within these organelles, CQ/HCQ increases the pH because of its biochemical behavior (O'Neill *et al*, 1998). Two main antiviral mechanisms have been identified: I) low‐pH‐depended inhibition of viral conformational changes that are essential for responsible for viral fusion, penetration, and uncoating; and II) inhibition/modification of post‐translational processing of viral glycoprotein’s in the trans‐GOLGI compartment and within endoplasmic vesicles (Randolph *et al*, 1990; Sieczkarski & Whittaker, 2002; Rolain *et al*, 2007). A third mechanism has been proposed, which is based on the immunomodulatory and anti‐inflammatory properties. Known effects related to this category include inhibition of intracellular Toll‐like receptors (such as TLR9), inhibitory effects on the cyclic‐AMP synthase pathway, and interference with major histocompatibility complex presentation (Rolain *et al*, 2007; Pal *et al*, 2020; Schrezenmeier & Dörner, 2020).

Antiviral activity of CQ/HCQ has been shown for viruses from several families and seems to cover a relatively broad spectrum (Rolain *et al*, 2007). Its antiviral mechanism is best explored in HIV where CQ induces modifications of the glycosylation pattern and amino acid charges from gp120 viral envelope protein, which may affect the immune escape mechanism of HIV (Savarino *et al*, 2004; Naarding *et al*, 2007). Although its clinical efficacy in HIV is not comparable to current antiretroviral drugs, several clinical studies have proven anti‐HIV effects of HCQ *in vivo* (Paton *et al*, 2002; Paton & Aboulhab, 2005). In CoVs different antiviral mechanisms of CQ/HCQ have been proposed including modifications to the viral spike glycoprotein (Gallagher *et al*, 1991; Vincent *et al*, 2005) and terminal post‐translational modification of ACE2‐receptor glycosylation, which might interfere with virus binding and consequent fusion (Li *et al*, 2003; Vincent *et al*, 2005). However, CQ/HCQ seems to elicit multiple effects on virus and host cell that ultimately inhibit viral replication. In a time‐of‐addition experiment, Liu *et al* (2020) confirmed that CQ/HCQ affects the viral life cycle both at cell entry and post‐entry stages. Intracellularly, CQ/HCQ showed to impair endosome maturation at intermediate stages of endocytosis, a crucial function for the transport of virions to its releasing site (Liu *et al*, 2020).

Activity against SARS‐CoV was demonstrated in Vero E6 cells with an EC_50_ of 8.8 µM (± 1.2) which approximates the plasma concentrations reached during treatment of acute malaria (Keyaerts *et al*, 2004). In SARS‐CoV‐2, CQ yielded EC_50_ of 1.13–1.38 µM and HCQ yielded 0.72–4.4 µM in RT–PCR‐based assays (Maisonnasse *et al*, 2020; Pizzorno *et al*, 2020; Wang *et al*, 2020a). Moreover, Liu *et al* 2020 directly compared *in vitro* efficacy of CQ with that of HCQ. By using Vero E6 cells that were exposed to SARS‐CoV‐2 with increasing multiplicities of infection (MOI), they found EC_50_ of 2.71–7.36 µM (CQ) and 4.06–12.96 µM (HCQ), respectively, in RT–PCR‐based assays (Liu *et al*, 2020). The authors concluded that HCQ is less potent compared with CQ. In contrast, Yao *et al* 2020 found lower EC_50_ for HCQ (0.72 µM) compared with CQ (5.47 µM) when using an MOI of 0.01 (the lowest MOI used by Liu *et al* 2020). They also included a physiologically based pharmacokinetic (PBPK) model of hydroxychloroquine concentrations in lung fluid. Based on this model, they predicted that an HCQ dose of 400 mg twice daily on day one followed by 200 mg twice daily seems to yield appropriate drug levels for treating COVID‐19 (Yao *et al*, 2020). These simulations are in line with an early pharmacokinetic study in children with rheumatic disease where 6‐6.5 mg/kg HCQ per day yielded serum levels of 1.4–1.5 µM in humans (Laaksonen *et al*, 1974). Chloroquine yielded plasma concentrations of 1–3 µM when applied with 3.6 mg/kg in another study (Wollheim *et al*, 1978). Tissue levels of both CQ and HCQ in animals were found to be 200–700 times higher than those in the plasma, including lung tissue (Popert, 1976). This suggests that sufficient drug concentrations may be reached at the site of infection in humans when using recommended doses but final evidence is lacking and pharmacokinetics can differ significantly in humans. However, based on these models, clinical trials have been conducted with maintenance doses of 400–600 HCQ mg daily.

Numerous clinical trials evaluating CQ/HCQ alone or in combination with additional drugs for the treatment of COVID‐19 were conducted or are still ongoing. At the time of writing, clinicaltrials.gov has registered 326 trials of CQ/HCQ in association with COVID‐19 treatment. However, most of the published results originate from observational studies or had a low enrollment size. Meanwhile, other trials have been halted due to emerging reports of HCQ‐induced cardiovascular events (Kalra *et al*, 2020; Kamp *et al*, 2020). Consequently, there has been discussion in the media and scientific community (Colafrancesco *et al*, 2020; Lenzer, 2020; Sharma, 2020). Some debate leading clinical finding will be mentioned briefly.

Early reports from China suggested a breakthrough in COVID‐19 treatment as results from more than 100 patients treated with CQ it could be concluded that it has a positive impact on disease course, viral clearance, lung images in contrast to control treatments (Gao *et al*, 2020). Although no clinical data were reported to support this hypothesis, HCQ was subsequently introduced in Chinese clinical guidelines. In a small Chinese randomized trial of 63 patients, HCQ seemed to reduce body temperature recovery time and the cough remission time (preprint: Chen *et al*, 2020b) and another small analysis of 26 patients treated with HCQ suggested effects on viral load reduction without any clinical implication (Gautret *et al*, 2020). Nevertheless, other studies could not identify any significant beneficial effect of CQ/HCQ (Mahévas *et al*, 2020; Chen *et al*, 2020a) including one observational study with 1376 patients enrolled (Geleris *et al*, 2020). One placebo‐controlled RCT sponsored by the NIH (NCT04332991) was recently halted after the forth interim analysis that included enrolled 479 patients suggested no beneficial effects of HCQ in COVID‐19 (NIH, 2020). Meanwhile, HCQ with or without azithromycin was tested in a non‐human primate model of SARS‐CoV‐2 infection where it showed no effects on viral load or clinical endpoints. In additional *in vitro* analyses published along with this animal study, anti‐SARS‐CoV‐2 activity of HCQ evident in Vero E6 cells could not be reproduced in human airway epithelial (HAE) cells which might explain diminished effects of CQ/HCQ *in vivo* (Maisonnasse *et al*, 2020). In conclusion, CQ/HCQ seems to be a broadly active antimicrobial agent that elicits multiple antiviral mechanisms and has potent *in vitro* efficacy against SARS‐CoV‐2 when tested in a Vero E6 cell model. However, clinical studies that demonstrate beneficial effects are lacking and available data mainly point toward a neglectable role in the clinical management of COVID‐19.

### Others

Myriads of clinical approved drugs have been tested regarding their activity against SARS‐CoV‐2 *in vitro*. Although other potent inhibitors could be identified, the clinical significance of those compounds is currently uncertain. Moreover, *in vivo* efficacy and specific MOA are largely unknown. Thus, we do not provide a detailed review of those compounds.

Choy *et al* 2020 reported on antiviral effects of homoharringtonine (omacetaxine mepesuccinate), a natural plant alkaloid used as a treatment of patients with chronic myeloid leukemia, and emetine, an antiprotozoal agent used in the treatment of amoebiasis. Both drugs block protein synthesis in eukaryotic cells (Gupta & Siminovitch, 1977; Gandhi *et al*, 2014). In a SARS‐CoV‐2 infection model with Vero E6 cells, the EC_50_ was 2.14–3.13 μM for homoharringtonine and 0.46−1.56 μM for emetine depending on antiviral assay (Choy *et al*, 2020). Wang *et al* 2020a found that nitazoxanide, a drug with broad‐spectrum antiparasitic and broad‐spectrum antiviral effects, inhibits SARS‐CoV‐2 at low‐micromolar concentration (EC_50_ = 2.12 μM) (Wang *et al*, 2020a). Recently, Pizzorno *et al* 2020 evaluated cyclosporine A, a calcineurin inhibitor used as an immunosuppressant medication, and berberine, an alkaloid found in several plants for anti‐SARS‐CoV‐2 activity. Cyclosporine has previously demonstrated antiviral activity against human coronavirus 229E (HCoV‐229E) and mouse hepatitis virus (MHV) but not SARS‐CoV (de Wilde *et al*, 2011). For berberine, inhibitory effects were shown against influenza, Chikungunya, and enterovirus 71 (Varghese *et al*, 2016). Both drugs were found to inhibit SARS‐CoV‐2 replication in Vero E6 cells significantly (EC_50_: cyclosporine A 3.05 μM; berberine 10.58 μM). Further studies have to clarify their potential role in COVID‐19 treatments.

## Concluding remarks and interpretation

In this comparative review, we focus on repurposed drugs with antiviral effects against SARS‐CoV‐2 in cell‐based assays as those substances offer great opportunities for a treatment early in the course of COVID‐19 by inhibition of viral replication and might be even suitable for preventive strategies as shown for neuraminidase inhibitors in case of influenza (Jefferson *et al*, 2014). In contrast, immunomodulatory drugs may be more beneficial in a later phase of infection, when the peak of viral replication has been reached and inflammatory processes dominate the pathophysiological process. This hypothesis is supported by the fact that repurposed immunomodulatory drugs like glucocorticoids seems to be beneficial in severe or critical COVID‐19 when used in a later phase after several days of symptomatic disease (preprint: Corral *et al*, 2020; Horby *et al*, 2020; Ramiro *et al*, 2020) but probably not within the first week after symptom onset (Horby *et al*, 2020).

Many substances were tested *in vitro* for their direct antiviral effects on SARS‐CoV‐2 replication or their ability to reduce cytopathologic effects in Vero E6 cells. However, to date only thirteen of them demonstrated any activity against SARS‐CoV‐2 (Table 1). Of repurposed entry and viral protease inhibitors, to date none has shown convincing evidence that support a clinical development as single agent against COVID‐19. Besides remdesivir which inhibits viral replication with an EC_50_ of 0.77–26.9 µM (depending on assay type, virus strain, and procedure of calculating), other nucleoside/nucleotide analogs that target the viral RdRp like favipiravir, penciclovir, or ribavirin were assessed but showed no or only weak activity against SARS‐CoV‐2. Inhibitors of viral protease were also investigated but only lopinavir had mentionable antiviral activity (EC_50_ 5.25–26.62 µM). Unfortunately, the combination of lopinavir and ritonavir did not show any clinical effects in a randomized controlled trial (Cao *et al*, 2020). The anti‐parasite drug CQ/HCQ was one of the most promising candidates against COVID‐19 based on preclinical studies but a clinical benefit could not be proven and a recently published *in vivo* study demonstrated no beneficial effects in a non‐human primate model of SARS‐CoV‐2 infection (Maisonnasse *et al*, 2020). Recent studies *in vitro* showed strong anti‐SARS‐CoV‐2 properties of compounds with different and partly unknown modes of antiviral action like nitazoxanide, cyclosporine A, emetine, and homoharringtonine. However, of those agents none have been readily assessed in animal models or clinical trials (Table 2).

**Table 2:** Published data from animal models and RCTs of repurposed drugs with proven anti‐SARS‐CoV‐2 efficacy *in vitro*

Repurposed drug	SARS‐CoV‐2 animal model	RCTs on COVID‐19 (identifier)	Main conclusions	References
Berberine	N/A	N/A	N/A	N/A
Chloroquine/Hydroxychloroquine		ChiCTR2000029559^a^	Reduced days of fever (2.2, SD 0.4 vs. 3.2, SD 1.3) and cough (2.0, SD 0.2 vs. 3.1, SD 1.5) in 62 patients	Chen *et al* (2020b)
		NCT04332991^a^	No additional benefit compared with placebo control for the treatment of COVID‐19 in hospitalized patients (n = 479; unpublished data)	NIH (2020)
	Cynomolgus macaque		No *in vivo* antiviral activity and no clinical efficacy, regardless of the timing of treatment initiation	Maisonnasse *et al* (2020)
Cyclosporine A	N/A	N/A	N/A	N/A
Emetine	N/A	N/A	N/A	N/A
Favipiravir	N/A	N/A	N/A	N/A
Homoharringtonine	N/A	N/A	N/A	N/A
Lopinavir (plus ritonavir)	N/A	ChiCTR2000029308	No clinical effect of 400/100 mg for 14 days	Cao *et al* (2020)
Nafamostat	N/A	N/A	N/A	N/A
Nitazoxanide	N/A	N/A	N/A	N/A
Penciclovir	N/A	N/A	N/A	N/A
Remdesivir		NCT04257656 P*re‐term suspended (N = 237)*	No significant clinical improvement (HR 1.23 [95% CI 0.87‐1.75])	Wang *et al* (2020)
		NCT04280705 (ACTT trial)	10 day course of RDV (200 mg d1, 100 mg 2‐10): 1. Reduction in time to recovery in adults hospitalized with COVID‐19 (hazard ratio: 1.31 [95% CI 1.12‐1.54]; *P* < 0.001). 2. Lower mortality rate in treatment group (8 % vs. 11.6%; *P* = 0.059)	Beigel *et al* (2020)
			
		NCT04292730^b^ (SIMPLE II)	No difference in clinical status distribution to placebo at day 11 after a 10 day course of RDV but better clinical status distribution after a 5 day course of RDV (odds ratio, 1.65 [95% CI 1.09‐2.48; *P* = 0.02). The result is of unclear clinical significance.	Spinner *et al* (2020)
	Rhesus macaque		RDV treated animals (*n* = 6) showed no clinical signs of disease, had lower lung virus titers and less lung tissue damage compared with the placebo group (*n* = 6)	Williamson *et al* (2020)
Ribavirin	N/A	N/A	N/A	N/A
Umifenovir (Arbidol)	N/A	N/A	N/A	N/A

SD, standard deviation; CI, 95% confidence interval.

^a^ Preliminary (not peer‐reviewed published) data.

^b^ Randomized controlled open‐label trial (no blinding was performed).

Therefore, remdesivir is the only antiviral drug that demonstrated efficacy in the preclinical and clinical setting. In the latter situation, it reduces time to recovery and may reduce mortality. A meta‐analysis which is available as preprint identified a statistically significant reduction in mortality (relative risk 0.69; [95% CI 0.49–0.99]) when pooling data of the two available RCTs (preprint: Alexander *et al*, 2020). Final results of the ACTT‐1 trial will provide more data to evaluate effects of RDV on mortality and virologic outcomes. In addition, a phase 1b/2a trial evaluating effects of RDV on viral load when administered by inhalation of an aerosolized solution is being planned (NCT04539262).

The relatively modest effect of the drug may be explainable by its virostatic mechanism of action and the fact that effects were studied after median 9 days of symptomatic disease (Beigel *et al*, 2020) while viral replication is dominating in the first week of infection (To *et al*, 2020; Wölfel *et al*, 2020; Zhou *et al*, 2020; He *et al*, 2020b). Early treatment with RDV was shown to be very effective in a rhesus macaque model of SARS‐CoV‐2 infection where it reduced clinical signs of infection, lung damage, and virus replication in lower respiratory tract specimen (Williamson *et al*, 2020). Based on these considerations, we hypothesize that treatment with RDV should therefore start early after symptom onset in the patient population with treatment indication. In contrast to other antiviral drugs, RDV is not available as oral formulation because of its poor bioavailability that is inherent to its phosphonate‐containing pro‐nucleoside design (Murakami *et al*, 2008; Pertusati *et al*, 2012). This is a major disadvantage as it precludes an early treatment initiation out of hospital. However, a clinical study that aims to evaluate multiple intravenous doses of RDV in an outpatient setting (NCT04501952) may increase our knowledge on its efficacy in early stages of COVID‐19.

The most successful antiviral therapies consist of combinations of antiviral drugs with different MOA’s as shown for HIV and HCV‐therapy. Here, this approach is necessary to prevent development of antiviral resistance during long‐term treatments. Nevertheless, combining of RDV with other antiviral or immunomodulatory agents may be a successful strategy to improve treatment outcomes.

## Author contributions

Conceptualization: JJM, AS; Original draft: JJM, AS, SJT; Visualization: AS; Project administration, Supervision of manuscript preparation, Validation: JJM; Review, Editing, Validation: JR, GF.

## Conflict of interest

J.J.M., A.S., S.J.T., and J.R. declare no potential conflicts of interest. G.F. has served as an advisor to Gilead Sciences and has conducted clinical research supported by Gilead Sciences.

## Pending issues

- Effects of RDV on mortality and virologic outcomes need to be addressed in the final publication of data from the ACTT‐1 trial and in subsequent meta‐analyses to substantiate the full potential of this antiviral drug.
- Safety and efficacy studies of RDV in combination with other antivirals or immunomodulatory drugs (including systemic corticosteroids) are needed. Hereby, drug–drug interactions must be taken into account as RDV interferes with several hepatic drug‐metabolizing enzymes.
- The optimal timing of RDV treatment is still unknown. Following studies should focus on patients in an early stage of COVID‐19.
- The poor oral bioavailability of RDV has many disadvantages and precludes a timely use out of hospital or in remote areas. Additional pharmacological efforts should be put into the development of an antiviral against SARS‐CoV‐2, which is orally available.

## For more information

- https://www.who.int/emergencies/diseases/novel‐coronavirus‐2019/global‐research‐on‐novel‐coronavirus‐2019‐ncov.
- https://clinicaltrials.gov/ct2/who_table.
- https://www.covid19treatmentguidelines.nih.gov/.
- https://www.bmj.com/content/370/bmj.m2980.

## References

[emmm202013105-bib-0001] Agostini ML , Andres EL , Sims AC , Graham RL , Sheahan TP , Lu X , Smith EC , Case JB , Feng JY , Jordan R *et al* (2018) Coronavirus susceptibility to the antiviral remdesivir (GS‐5734) is mediated by the viral polymerase and the proofreading exoribonuclease. MBio 9: e00221‐e218 29511076 10.1128/mBio.00221-18 PMC5844999

[emmm202013105-bib-0002] Alexander PE , Piticaru J , Lewis K , Aryal K , Thomas P , Szczeklik W , Fronczek J , Polok K , Alhazzani W , Mammen M (2020) Remdesivir use in patients with coronavirus COVID‐19 disease: a systematic review and meta‐analysis. medRxiv 10.1101/2020.05.23.20110932 [PREPRINT]

[emmm202013105-bib-0003] Anand K , Ziebuhr J , Wadhwani P , Mesters JR , Hilgenfeld R (2003) Coronavirus main proteinase (3CLpro) structure: basis for design of anti‐SARS drugs. Science 300: 1763–1767 12746549 10.1126/science.1085658

[emmm202013105-bib-0004] Antalis TM , Bugge TH , Wu Q (2011) Membrane‐anchored serine proteases in health and disease. Prog Mol Biol Transl Sci 99: 1–50 21238933 10.1016/B978-0-12-385504-6.00001-4 PMC3697097

[emmm202013105-bib-0005] Banerjee A , Nasir JA , Budylowski P , Yip L , Aftanas P , Christie N , Ghalami A , Baid K , Raphenya AR , Hirota JA *et al* (2020) Isolation, sequence, infectivity, and replication kinetics of severe acute respiratory syndrome coronavirus 2. Emerg Infect Dis 26(9): 2054–2063 32558639 10.3201/eid2609.201495 PMC7454076

[emmm202013105-bib-0006] Beigel JH , Tomashek KM , Dodd LE , Mehta AK , Zingman BS , Kalil AC , … Kline S *et al* (2020) Remdesivir for the treatment of covid‐19 ‐ preliminary report. N Engl J Med 383: 992–993 10.1056/NEJMc2022236 32649078

[emmm202013105-bib-0007] Boriskin YS , Leneva IA , Pécheur EI , Polyak SJ (2008) Arbidol: a broad‐spectrum antiviral compound that blocks viral fusion. Curr Med Chem 15: 997–1005 18393857 10.2174/092986708784049658

[emmm202013105-bib-0008] Bosch BJ , van der Zee R , de Haan CA , Rottier PJ (2003) The coronavirus spike protein is a class I virus fusion protein: structural and functional characterization of the fusion core complex. J Virol 77: 8801–8811 12885899 10.1128/JVI.77.16.8801-8811.2003 PMC167208

[emmm202013105-bib-0009] Brown AJ , Won JJ , Graham RL , Dinnon KH , Sims AC , Feng JY , Cihlar T , Denison MR , Baric RS , Sheahan TP (2019) Broad spectrum antiviral remdesivir inhibits human endemic and zoonotic deltacoronaviruses with a highly divergent RNA dependent RNA polymerase. Antiviral Res 169: 104541 31233808 10.1016/j.antiviral.2019.104541 PMC6699884

[emmm202013105-bib-0010] Burkard C , Verheije MH , Wicht O , van Kasteren SI , van Kuppeveld FJ , Haagmans BL , Pelkmans L , Rottier PJM , Bosch BJ , de Haan CAM (2014) Coronavirus cell entry occurs through the endo‐/lysosomal pathway in a proteolysis‐dependent manner. PLoS Pathog 10: e1004502 25375324 10.1371/journal.ppat.1004502 PMC4223067

[emmm202013105-bib-0011] Cai Q , Yang M , Liu D , Chen J , Shu D , Xia J , Liao X , Gu Y , Cai Q , Yang Y *et al* (2020) Experimental treatment with favipiravir for COVID‐19: an open‐label control study. Engineering (Beijing). 10.1016/j.eng.2020.03.007 PMC7185795 32346491

[emmm202013105-bib-0012] Cao B , Wang Y , Wen D , Liu W , Wang J , Fan G , Ruan L , Song B , Cai Y , Wei M *et al* (2020) A trial of lopinavir‐ritonavir in adults hospitalized with severe Covid‐19. N Engl J Med 382: 1787–1799 32187464 10.1056/NEJMoa2001282 PMC7121492

[emmm202013105-bib-0013] Chan JF‐W , Yao Y , Yeung M‐L , Deng W , Bao L , Jia L , Li F , Xiao C , Gao H , Yu P *et al* (2015) Treatment with Lopinavir/Ritonavir or interferon‐β1b improves outcome of MERS‐CoV infection in a nonhuman primate model of common marmoset. J Infect Dis 212: 1904–1913 26198719 10.1093/infdis/jiv392 PMC7107395

[emmm202013105-bib-0014] Chang JH , Lee IS , Kim HK , Cho YK , Park JM , Kim SW , Choi M‐G , Chung IS (2009) Nafamostat for Prophylaxis against Post‐Endoscopic Retrograde Cholangiopancreatography Pancreatitis Compared with Gabexate. Gut Liv 3: 205–210 10.5009/gnl.2009.3.3.205 PMC2852705 20431747

[emmm202013105-bib-0015] Chen J , Liu D , Liu L , Liu P , Xu Q , Xia L , Ling Y , Huang D , Song S , Zhang D *et al* (2020a) A pilot study of hydroxychloroquine in treatment of patients with moderate COVID‐19. Zhejiang Da Xue Xue Bao Yi Xue Ban 49: 215–219 32391667 10.3785/j.issn.1008-9292.2020.03.03 PMC8800713

[emmm202013105-bib-0016] Chen Z , Hu J , Zhang Z , Jiang S , Han S , Yan D , Zhuang R , Hu B , Zhang Z (2020b) Efficacy of hydroxychloroquine in patients with COVID‐19: results of a randomized clinical trial. medRxiv 10.1101/2020.03.22.20040758 [PREPRINT]

[emmm202013105-bib-0017] Cho A , Saunders OL , Butler T , Zhang L , Xu J , Vela JE , Feng JY , Ray AS , Kim CU (2012) Synthesis and antiviral activity of a series of 1'‐substituted 4‐aza‐7,9‐dideazaadenosine C‐nucleosides. Bioorg Med Chem Lett 22: 2705–2707 22446091 10.1016/j.bmcl.2012.02.105 PMC7126871

[emmm202013105-bib-0018] Choy KT , Wong AY , Kaewpreedee P , Sia SF , Chen D , Hui KPY , Chu DKW , Chan MCW , Cheung PP , Huang X *et al* (2020) Remdesivir, lopinavir, emetine, and homoharringtonine inhibit SARS‐CoV‐2 replication in vitro. Antiviral Res 178: 104786 32251767 10.1016/j.antiviral.2020.104786 PMC7127386

[emmm202013105-bib-0019] Cinatl J , Morgenstern B , Bauer G , Chandra P , Rabenau H , Doerr HW (2003) Glycyrrhizin, an active component of liquorice roots, and replication of SARS‐associated coronavirus. Lancet 361: 2045–2046 12814717 10.1016/S0140-6736(03)13615-X PMC7112442

[emmm202013105-bib-0020] Colafrancesco S , Scrivo R , Barbati C , Conti F , Priori R (2020) Targeting the immune system for pulmonary inflammation and cardiovascular complications in COVID‐19 patients. Front Immunol 11: 1439 32655577 10.3389/fimmu.2020.01439 PMC7324709

[emmm202013105-bib-0021] Corman VM , Eckerle I , Bleicker T , Zaki A , Landt O , Eschbach‐Bludau M , van Boheemen S , Gopal R , Ballhause M , Bestebroer TM *et al* (2012) Detection of a novel human coronavirus by real‐time reverse‐transcription polymerase chain reaction. Euro Surveill 17: 20285 23041020 10.2807/ese.17.39.20285-en

[emmm202013105-bib-0022] Corral L , Bahamonde A , Arnaiz delas Revillas F , Gomez‐Barquero J , Abadia‐Otero J , Garcia‐Ibarbia C , Mora V , Cerezo‐Hernandez A , Hernandez JL , Lopez‐Muniz G *et al* (2020) GLUCOCOVID: A controlled trial of methylprednisolone in adults hospitalized with COVID‐19 pneumonia. medRxiv 10.1101/2020.06.17.20133579 [PREPRINT] PMC7854876 33534047

[emmm202013105-bib-0023] Crance JM , Scaramozzino N , Jouan A , Garin D (2003) Interferon, ribavirin, 6‐azauridine and glycyrrhizin: antiviral compounds active against pathogenic flaviviruses. Antiviral Res 58: 73–79 12719009 10.1016/s0166-3542(02)00185-7

[emmm202013105-bib-0024] de Haan CA , Rottier PJ (2005) Molecular interactions in the assembly of coronaviruses. Adv Virus Res 64: 165–230 16139595 10.1016/S0065-3527(05)64006-7 PMC7112327

[emmm202013105-bib-0025] De Meyer S , Bojkova D , Cinatl J , Van Damme E , Buyck C , Van Loock M , Woodfall B , Ciesek S (2020) Lack of antiviral activity of darunavir against SARS‐CoV‐2. Int J Infect Dis 97: 7–10 32479865 10.1016/j.ijid.2020.05.085 PMC7258847

[emmm202013105-bib-0026] de Wilde AH , Jochmans D , Posthuma CC , ZevenhoVen‐Dobbe JC , van Nieuwkoop S , Bestebroer TM , van Den Hoogen BG , Neyts J , Snijder EJ (2014) Screening of an FDA‐approved compound library identifies four small‐molecule inhibitors of Middle East respiratory syndrome coronavirus replication in cell culture. Antimicrob Agents Chemother 58: 4875–4884 24841269 10.1128/AAC.03011-14 PMC4136071

[emmm202013105-bib-0027] de Wilde AH , Zevenhoven‐Dobbe JC , van der Meer Y , Thiel V , Narayanan K , Makino S , Snijder EJ , van Hemert MJ (2011) Cyclosporin A inhibits the replication of diverse coronaviruses. J Gen Virol 92: 2542–2548 21752960 10.1099/vir.0.034983-0 PMC3352363

[emmm202013105-bib-0028] de Wit E , Feldmann F , Cronin J , Jordan R , Okumura A , Thomas T , Scott D , Cihlar T , Feldmann H (2020) Prophylactic and therapeutic remdesivir (GS‐5734) treatment in the rhesus macaque model of MERS‐CoV infection. Proc Natl Acad Sci USA 117: 6771–6776 32054787 10.1073/pnas.1922083117 PMC7104368

[emmm202013105-bib-0029] Dey SK , Saini M , Dhembla C , Bhatt S , Rajesh AS , Anand V , Kundu S (2020) Suramin, Penciclovir and Anidulafungin bind nsp12, which governs the RNA‐dependent‐RNA polymerase activity of SARS‐CoV‐2, with higher interaction energy than Remdesivir, indicating potential in the treatment of Covid‐19 infection. OSF Preprints 10.31219/osf.io/urxwh [PREPRINT]

[emmm202013105-bib-0030] Drosten C , Günther S , Preiser W , van der Werf S , Brodt HR , Becker S , Rabenau H , Panning M , Kolesnikova L , Fouchier RA *et al* (2003) Identification of a novel coronavirus in patients with severe acute respiratory syndrome. N Engl J Med 348: 1967–1976 12690091 10.1056/NEJMoa030747

[emmm202013105-bib-0031] Fan Z , Chen L , Li J , Cheng X , Yang J , Tian C , Zhang Y , Huang S , Liu Z , Cheng J (2020) Clinical features of COVID‐19‐related liver functional abnormality. Clin Gastroenterol Hepatol 18: 1561–1566 32283325 10.1016/j.cgh.2020.04.002 PMC7194865

[emmm202013105-bib-0032] Food and Drug Administration (FDA) (2020) Fact Sheet For Health Care Providers Emergency Use Authorization (Eua) Of Veklury® (remdesivir), Available at: www.fda.gov/media/137566/download Accessed 12.08.2020

[emmm202013105-bib-0033] Fehr AR , Perlman S (2015) Coronaviruses: an overview of their replication and pathogenesis. Methods Mol Biol 1282: 1–23 25720466 10.1007/978-1-4939-2438-7_1 PMC4369385

[emmm202013105-bib-0034] Furuta Y , Gowen BB , Takahashi K , Shiraki K , Smee DF , Barnard DL (2013) Favipiravir (T‐705), a novel viral RNA polymerase inhibitor. Antiviral Res 100: 446–454 24084488 10.1016/j.antiviral.2013.09.015 PMC3880838

[emmm202013105-bib-0035] Furuta Y , Takahashi K , Fukuda Y , Kuno M , Kamiyama T , Kozaki K , Nomura N , Egawa H , Minami S , Watanabe Y *et al* (2002) In vitro and in vivo activities of anti‐influenza virus compound T‐705. Antimicrob Agents Chemother 46: 977–981 11897578 10.1128/AAC.46.4.977-981.2002 PMC127093

[emmm202013105-bib-0036] Gallagher TM , Escarmis C , Buchmeier MJ (1991) Alteration of the pH dependence of coronavirus‐induced cell fusion: effect of mutations in the spike glycoprotein. J Virol 65: 1916–1928 1848311 10.1128/jvi.65.4.1916-1928.1991 PMC240014

[emmm202013105-bib-0037] Gandhi V , Plunkett W , Cortes JE (2014) Omacetaxine: a protein translation inhibitor for treatment of chronic myelogenous leukemia. Clin Cancer Res 20: 1735–1740 24501394 10.1158/1078-0432.CCR-13-1283 PMC4048124

[emmm202013105-bib-0038] Gao J , Tian Z , Yang X (2020) Breakthrough: Chloroquine phosphate has shown apparent efficacy in treatment of COVID‐19 associated pneumonia in clinical studies. Biosci Trends 14: 72–73 32074550 10.5582/bst.2020.01047

[emmm202013105-bib-0039] Gautret P , Lagier JC , Parola P , Hoang VT , Meddeb L , Mailhe M , Doudier B , Courjon J , Giordanengo V , Vieira VE *et al* (2020) Hydroxychloroquine and azithromycin as a treatment of COVID‐19: results of an open‐label non‐randomized clinical trial. Int J Antimicrob Agents 105949 10.1016/j.ijantimicag.2020.105949 PMC7102549 32205204

[emmm202013105-bib-0040] Geleris J , Sun Y , Platt J , Zucker J , Baldwin M , Hripcsak G , Labella A , Manson DK , Kubin C , Barr RG *et al* (2020) Observational Study of Hydroxychloroquine in Hospitalized Patients with Covid‐19. N Engl J Med 382: 2411–2418 32379955 10.1056/NEJMoa2012410 PMC7224609

[emmm202013105-bib-0041] Gilead Sciences (2020) Remdesivir (GS‐5734®) Investigator's Brochure Version 5.0. Foster City, CA, Gilead Sciences, Inc., 115–116.

[emmm202013105-bib-0042] Goldman JD , Lye DCB , Hui DS , Marks KM , Bruno R , Montejano R , Spinner CD , Galli M , Ahn MY , Nahass RG *et al* (2020) Remdesivir for 5 or 10 days in patients with severe Covid‐19. N Engl J Med 10.1056/NEJMoa2015301. PMC7377062 32459919

[emmm202013105-bib-0043] Gordon CJ , Tchesnokov EP , Feng JY , Porter DP , Götte M (2020a) The antiviral compound remdesivir potently inhibits RNA‐dependent RNA polymerase from Middle East respiratory syndrome coronavirus. J Biol Chem 295: 4773–4779 32094225 10.1074/jbc.AC120.013056 PMC7152756

[emmm202013105-bib-0044] Gordon CJ , Tchesnokov EP , Woolner E , Perry JK , Feng JY , Porter DP , Götte M (2020b) Remdesivir is a direct‐acting antiviral that inhibits RNA‐dependent RNA polymerase from severe acute respiratory syndrome coronavirus 2 with high potency. J Biol Chem 295: 6785–6797 32284326 10.1074/jbc.RA120.013679 PMC7242698

[emmm202013105-bib-0045] Grein J , Ohmagari N , Shin D , Diaz G , Asperges E , Castagna A , Feldt T , Green G , Green ML , Lescure FX *et al* (2020) Compassionate use of remdesivir for patients with severe Covid‐19. N Engl J Med. 382(24): 2327–2336 32275812 10.1056/NEJMoa2007016 PMC7169476

[emmm202013105-bib-0046] Guan Y , Zheng BJ , He YQ , Liu XL , Zhuang ZX , Cheung CL , Luo SW , Li PH , Zhang LJ , Guan YJ *et al* (2003) Isolation and characterization of viruses related to the SARS coronavirus from animals in Southern China. Science 302: 276–278 12958366 10.1126/science.1087139

[emmm202013105-bib-0047] Gupta RS , Siminovitch L (1977) The molecular basis of emetine resistance in Chinese hamster ovary cells: alteration in the 40S ribosomal subunit. Cell 10: 61–66 837444 10.1016/0092-8674(77)90140-4

[emmm202013105-bib-0048] He J , Hu L , Huang X , Wang C , Zhang Z , Wang Y , Zhang D , Ye W (2020a) Potential of coronavirus 3C‐like protease inhibitors for the development of new anti‐SARS‐CoV‐2 drugs: Insights from structures of protease and inhibitors. Int J Antimicrob Agents 56(2): 106055 32534187 10.1016/j.ijantimicag.2020.106055 PMC7286838

[emmm202013105-bib-0049] He X , Lau EHY , Wu P , Deng X , Wang J , Hao X , Lau YC , Wong JY , Guan Y , Tan X *et al* (2020b) Temporal dynamics in viral shedding and transmissibility of COVID‐19. Nat Med 26: 672–675 32296168 10.1038/s41591-020-0869-5

[emmm202013105-bib-0050] Hoffmann M , Kleine‐Weber H , Schroeder S , Krüger N , Herrler T , Erichsen S , Schiergens TS , Herrler G , Wu NH , Nitsche A *et al* (2020a) SARS‐CoV‐2 cell entry depends on ACE2 and TMPRSS2 and is blocked by a clinically proven protease inhibitor. Cell 181: 271–280. 32142651 10.1016/j.cell.2020.02.052 PMC7102627

[emmm202013105-bib-5100] Hoffmann M , Schroeder S , Kleine‐Weber H , Müller MA , Drosten C , Pöhlmann S (2020b) Nafamostat mesylate blocks activation of SARS‐CoV‐2: new treatment option for COVID‐19. Antimicrob Agents Chemother 64: e00754‐20 32312781 10.1128/AAC.00754-20 PMC7269515

[emmm202013105-bib-0051] Horby P , Lim WS , Emberson JR , Mafham M , Bell JL , Linsell L , Staplin N , Brightling C , Ustianowski A , Elmahi E *et al* (2020) Dexamethasone in hospitalized patients with Covid‐19 – preliminary report. N Engl J Med 10.1056/NEJMoa2021436 PMC7383595 32678530

[emmm202013105-bib-0052] Huang D , Yu H , Wang T , Yang H , Yao R , Liang Z (2020) Efficacy and safety of umifenovir for coronavirus disease 2019 (COVID‐19): A systematic review and meta‐analysis. J Med Virol 192: E734–E744 10.1002/jmv.26256 PMC7361300 32617989

[emmm202013105-bib-0053] Iwata‐Yoshikawa N , Okamura T , Shimizu Y , Hasegawa H , Takeda M , Nagata N (2019) TMPRSS2 contributes to virus spread and immunopathology in the airways of murine models after coronavirus infection. J Virol 93: e01815‐18 30626688 10.1128/JVI.01815-18 PMC6401451

[emmm202013105-bib-0054] Jefferson T , Jones MA , Doshi P , Del Mar CB , Hama R , Thompson MJ , Spencer EA , Onakpoya I , Mahtani KR , Nunan D *et al* (2014) Neuraminidase inhibitors for preventing and treating influenza in healthy adults and children. Cochrane Database Syst Rev 2014: Cd008965 10.1002/14651858.CD008965.pub4 PMC6464969 24718923

[emmm202013105-bib-0055] Jochmans D , van Nieuwkoop S , Smits SL , Neyts J , Fouchier RA , van den Hoogen BG (2016) Antiviral activity of favipiravir (T‐705) against a broad range of paramyxoviruses in vitro and against human metapneumovirus in hamsters. Antimicrob Agents Chemother 60: 4620–4629 27185803 10.1128/AAC.00709-16 PMC4958190

[emmm202013105-bib-0056] Johns Hopkins University (JHU) (2020). COVID‐19 Dashboard by the Center for Systems Science and Engineering (CSSE). Available at: http://isanddata.maps.arcgis.com/apps/opsdashboard/index.html#/bda7594740fd40299423467b48e9ecf6. Accessed September, 22 2020

[emmm202013105-bib-0057] Jordheim LP , Durantel D , Zoulim F , Dumontet C (2013) Advances in the development of nucleoside and nucleotide analogues for cancer and viral diseases. Nat Rev Drug Discov 12: 447–464 23722347 10.1038/nrd4010

[emmm202013105-bib-0058] Kalra RS , Tomar D , Meena AS , Kandimalla R (2020) SARS‐CoV‐2, ACE2, and hydroxychloroquine: cardiovascular complications, therapeutics, and clinical readouts in the current settings. Pathogens 9: 546 10.3390/pathogens9070546 PMC7400328 32645974

[emmm202013105-bib-0059] Kamp TJ , Hamdan MH , January CT (2020) Chloroquine or hydroxychloroquine for COVID‐19: is cardiotoxicity a concern? J Am Heart Assoc 9: e016887 32463308 10.1161/JAHA.120.016887 PMC7429067

[emmm202013105-bib-0060] Kawase M , Shirato K , van der Hoek L , Taguchi F , Matsuyama S (2012) Simultaneous treatment of human bronchial epithelial cells with serine and cysteine protease inhibitors prevents severe acute respiratory syndrome coronavirus entry. J Virol 86: 6537–6545 22496216 10.1128/JVI.00094-12 PMC3393535

[emmm202013105-bib-0061] Keyaerts E , Vijgen L , Maes P , Neyts J , Van Ranst M (2004) In vitro inhibition of severe acute respiratory syndrome coronavirus by chloroquine. Biochem Biophys Res Comm 323: 264–268 15351731 10.1016/j.bbrc.2004.08.085 PMC7092815

[emmm202013105-bib-0062] Ksiazek TG , Erdman D , Goldsmith CS , Zaki SR , Peret T , Emery S , Tong S , Urbani C , Comer JA , Lim W *et al* (2003) A novel coronavirus associated with severe acute respiratory syndrome. N Engl J Med 348: 1953–1966 12690092 10.1056/NEJMoa030781

[emmm202013105-bib-0063] Laaksonen AL , Koskiahde V , Juva K (1974) Dosage of antimalarial drugs for children with juvenile rheumatoid arthritis and systemic lupus erythematosus. A clinical study with determination of serum concentrations of chloroquine and hydroxychloroquine. Scand J Rheumatol 3: 103–108 4608161 10.3109/03009747409115809

[emmm202013105-bib-0064] Lei F , Liu YM , Zhou F , Qin JJ , Zhang P , Zhu L , … Ouyang S *et al* (2020) Longitudinal association between markers of liver injury and mortality in COVID‐19 in China. Hepatology 72: 389–398 32359177 10.1002/hep.31301 PMC7267515

[emmm202013105-bib-0065] Leneva IA , Russell RJ , Boriskin YS , Hay AJ (2009) Characteristics of arbidol‐resistant mutants of influenza virus: implications for the mechanism of anti‐influenza action of arbidol. Antiviral Res 81: 132–140 19028526 10.1016/j.antiviral.2008.10.009

[emmm202013105-bib-0066] Lenzer J (2020) Covid‐19: US gives emergency approval to hydroxychloroquine despite lack of evidence. BMJ 369: m1335 32238355 10.1136/bmj.m1335

[emmm202013105-bib-0067] Li W , Moore MJ , Vasilieva N , Sui J , Wong SK , Berne MA , Somasundaran M , Sullivan JL , Luzuriaga K , Greenough TC *et al* (2003) Angiotensin‐converting enzyme 2 is a functional receptor for the SARS coronavirus. Nature 426: 450–454 14647384 10.1038/nature02145 PMC7095016

[emmm202013105-bib-0068] Li W , Shi Z , Yu M , Ren W , Smith C , Epstein JH , Wang H , Crameri G , Hu Z , Zhang H *et al* (2005) Bats are natural reservoirs of SARS‐like coronaviruses. Science 310: 676–679 16195424 10.1126/science.1118391

[emmm202013105-bib-0069] Liu J , Cao R , Xu M , Wang X , Zhang H , Hu H , … Wang M (2020) Hydroxychloroquine, a less toxic derivative of chloroquine, is effective in inhibiting SARS‐CoV‐2 infection in vitro. Cell Discov 6: 16 32194981 10.1038/s41421-020-0156-0 PMC7078228

[emmm202013105-bib-0070] Lo MK , Jordan R , Arvey A , Sudhamsu J , Shrivastava‐Ranjan P , Hotard AL , Flint M , McMullan LK , Siegel D , Clarke MO *et al* (2017) GS‐5734 and its parent nucleoside analog inhibit Filo‐, Pneumo‐, and Paramyxoviruses. Sci Rep 7: 43395 28262699 10.1038/srep43395 PMC5338263

[emmm202013105-bib-0071] Mahévas M , Tran VT , Roumier M , Chabrol A , Paule R , Guillaud C , Fois E , Lepeule R , Szwebel TA , Lescure FX *et al* (2020) Clinical efficacy of hydroxychloroquine in patients with covid‐19 pneumonia who require oxygen: observational comparative study using routine care data. BMJ 369: m1844 32409486 10.1136/bmj.m1844 PMC7221472

[emmm202013105-bib-0072] Maisonnasse P , Guedj J , Contreras V , Behillil S , Solas C , Marlin R , Naninck T , Pizzorno A , Lemaitre J , Gonçalves A *et al* (2020) Hydroxychloroquine use against SARS‐CoV‐2 infection in non‐human primates. Nature 585: 584–587 32698191 10.1038/s41586-020-2558-4

[emmm202013105-bib-0073] McChesney EW (1983) Animal toxicity and pharmacokinetics of hydroxychloroquine sulfate. Am J Med 75: 11–18 6408923 10.1016/0002-9343(83)91265-2

[emmm202013105-bib-0074] Mohd HA , Al‐Tawfiq JA , Memish ZA (2016) Middle east respiratory syndrome coronavirus (MERS‐CoV) origin and animal reservoir. Virol J 13: 87 27255185 10.1186/s12985-016-0544-0 PMC4891877

[emmm202013105-bib-0075] Mulangu S , Dodd LE , Davey Jr RT , Tshiani Mbaya O , Proschan M , Mukadi D , Lusakibanza Manzo M , Nzolo D , Tshomba Oloma A , Ibanda A *et al* (2019) A randomized, controlled trial of ebola virus disease therapeutics. N Engl J Med 381: 2293–2303 31774950 10.1056/NEJMoa1910993 PMC10680050

[emmm202013105-bib-0076] Murakami E , Niu C , Bao H , Micolochick Steuer HM , Whitaker T , Nachman T , Sofia MA , Wang P , Otto MJ , Furman PA (2008) The mechanism of action of beta‐D‐2'‐deoxy‐2'‐fluoro‐2'‐C‐methylcytidine involves a second metabolic pathway leading to beta‐D‐2'‐deoxy‐2'‐fluoro‐2'‐C‐methyluridine 5'‐triphosphate, a potent inhibitor of the hepatitis C virus RNA‐dependent RNA polymerase. Antimicrob Agents Chemother 52: 458–464 17999967 10.1128/AAC.01184-07 PMC2224766

[emmm202013105-bib-0077] Naarding MA , Baan E , Pollakis G , Paxton WA (2007) Effect of chloroquine on reducing HIV‐1 replication in vitro and the DC‐SIGN mediated transfer of virus to CD4+ T‐lymphocytes. Retrovirology 4: 6 17263871 10.1186/1742-4690-4-6 PMC1796897

[emmm202013105-bib-0078] National Institutes of Health (NIH) (2020). NIH halts clinical trial of hydroxychloroquine. Press release available at: www.nih.gov/news‐events/news‐releases/nih‐halts‐clinical‐trial‐hydroxychloroquine. Accessed June, 28 2020

[emmm202013105-bib-0079] O'Neill PM , Bray PG , Hawley SR , Ward SA , Park BK (1998) 4‐Aminoquinolines–past, present, and future: a chemical perspective. Pharmacol Ther 77: 29–58 9500158 10.1016/s0163-7258(97)00084-3

[emmm202013105-bib-0080] Ogando NS , Dalebout TJ , Zevenhoven‐Dobbe JC , Limpens RWAL , van der Meer Y , Caly L , Druce J , de Vries JJC , Kikkert M , Bárcena M *et al* (2020) SARS‐coronavirus‐2 replication in Vero E6 cells: replication kinetics, rapid adaptation and cytopathology. J Gen Virol. 101: 925–940 32568027 10.1099/jgv.0.001453 PMC7654748

[emmm202013105-bib-0081] Ou X , Liu Y , Lei X , Li P , Mi D , Ren L , Guo L , Guo R , Chen T , Hu J *et al* (2020) Characterization of spike glycoprotein of SARS‐CoV‐2 on virus entry and its immune cross‐reactivity with SARS‐CoV. Nat Commun 11: 1620 32221306 10.1038/s41467-020-15562-9 PMC7100515

[emmm202013105-bib-0082] Pal A , Pawar A , Goswami K , Sharma P , Prasad R (2020) Hydroxychloroquine and Covid‐19: a cellular and molecular biology based update. Indian J Clin Biochem 35: 274–284 32641874 10.1007/s12291-020-00900-x PMC7286553

[emmm202013105-bib-0083] Paton NI , Aboulhab J (2005) Hydroxychloroquine, hydroxyurea and didanosine as initial therapy for HIV‐infected patients with low viral load: safety, efficacy and resistance profile after 144 weeks. HIV Med 6: 13–20 15670247 10.1111/j.1468-1293.2005.00259.x

[emmm202013105-bib-0084] Paton NI , Aboulhab J , Karim F (2002) Hydroxychloroquine, hydroxycarbamide, and didanosine as economic treatment for HIV‐1. Lancet 359: 1667–1668 12020529 10.1016/S0140-6736(02)08557-4

[emmm202013105-bib-0085] Peiris JS , Lai ST , Poon LL , Guan Y , Yam LY , Lim W , Nicholls J , Yee WK , Yan WW , Cheung MT *et al* (2003) Coronavirus as a possible cause of severe acute respiratory syndrome. Lancet 361: 1319–1325 12711465 10.1016/S0140-6736(03)13077-2 PMC7112372

[emmm202013105-bib-0086] Pertusati F , Serpi M , McGuigan C (2012) Medicinal chemistry of nucleoside phosphonate prodrugs for antiviral therapy. Antivir Chem Chemother 22: 181–203 22182785 10.3851/IMP2012

[emmm202013105-bib-0087] Pizzorno A , Padey B , Dubois J , Julien T , Traversier A , Dulière V , Brun P , Lina B , Rosa‐Calatrava M , Terrier O (2020) In vitro evaluation of antiviral activity of single and combined repurposable drugs against SARS‐CoV‐2. Antiviral Res 181: 104878 32679055 10.1016/j.antiviral.2020.104878 PMC7361110

[emmm202013105-bib-0088] Popert AJ (1976) Chloroquine: a review. Rheumatol Rehabil 15: 235–238 968356 10.1093/rheumatology/15.3.235

[emmm202013105-bib-0089] Ramiro S , Mostard RLM , Magro‐Checa C , van Dongen CMP , Dormans T , Buijs J , Gronenschild M , de Kruif MD , van Haren EHJ , van Kraaij T *et al* (2020) Historically controlled comparison of glucocorticoids with or without tocilizumab versus supportive care only in patients with COVID‐19‐associated cytokine storm syndrome: results of the CHIC study. Ann Rheum Dis 79: 1143–1151 32719045 10.1136/annrheumdis-2020-218479 PMC7456552

[emmm202013105-bib-0090] Ramsey ML , Nuttall J , Hart PA (2019) A phase 1/2 trial to evaluate the pharmacokinetics, safety, and efficacy of NI‐03 in patients with chronic pancreatitis: study protocol for a randomized controlled trial on the assessment of camostat treatment in chronic pancreatitis (TACTIC). Trials 20: 501 31412955 10.1186/s13063-019-3606-y PMC6694471

[emmm202013105-bib-0091] Randolph VB , Winkler G , Stollar V (1990) Acidotropic amines inhibit proteolytic processing of flavivirus prM protein. Virology 174: 450–458 2154882 10.1016/0042-6822(90)90099-d

[emmm202013105-bib-0092] Rau JC , Beaulieu LM , Huntington JA , Church FC (2007) Serpins in thrombosis, hemostasis and fibrinolysis. J Thromb Haemost 5(Suppl 1): 102–115 10.1111/j.1538-7836.2007.02516.x PMC2670448 17635716

[emmm202013105-bib-0093] Rolain JM , Colson P , Raoult D (2007) Recycling of chloroquine and its hydroxyl analogue to face bacterial, fungal and viral infections in the 21st century. Int J Antimicrob Agents 30: 297–308 17629679 10.1016/j.ijantimicag.2007.05.015 PMC7126847

[emmm202013105-bib-0094] Sangawa H , Komeno T , Nishikawa H , Yoshida A , Takahashi K , Nomura N , Furuta Y (2013) Mechanism of action of T‐705 ribosyl triphosphate against influenza virus RNA polymerase. Antimicrob Agents Chemother 57: 5202–5208 23917318 10.1128/AAC.00649-13 PMC3811313

[emmm202013105-bib-0095] Savarino A , Lucia MB , Rastrelli E , Rutella S , Golotta C , Morra E , Tamburrini E , Perno CF , Boelaert JR , Sperber K *et al* (2004) Anti‐HIV effects of chloroquine: inhibition of viral particle glycosylation and synergism with protease inhibitors. J Acquir Immune Defic Syndr 35: 223–232 15076236 10.1097/00126334-200403010-00002

[emmm202013105-bib-0096] Schrezenmeier E , Dörner T (2020) Mechanisms of action of hydroxychloroquine and chloroquine: implications for rheumatology. Nat Rev Rheumatol 16: 155–166 32034323 10.1038/s41584-020-0372-x

[emmm202013105-bib-0097] Seley‐Radtke KL , Yates MK (2018) The evolution of nucleoside analogue antivirals: A review for chemists and non‐chemists. Part 1: Early structural modifications to the nucleoside scaffold. Antiviral Res 154: 66–86 29649496 10.1016/j.antiviral.2018.04.004 PMC6396324

[emmm202013105-bib-0098] Sham HL , Kempf DJ , Molla A , Marsh KC , Kumar GN , Chen CM , Kati W , Stewart K , Lal R , Hsu A *et al* (1998) ABT‐378, a highly potent inhibitor of the human immunodeficiency virus protease. Antimicrob Agents Chemother 42: 3218–3224 9835517 10.1128/aac.42.12.3218 PMC106025

[emmm202013105-bib-0099] Shannon A , Selisko B , Le N , Huchting J , Touret F , Piorkowski G , Fattorini V , Ferron F , Decroly E , Meier C *et al* (2020) Favipiravir strikes the SARS‐CoV‐2 at its Achilles heel, the RNA polymerase. bioRxiv 10.1101/2020.05.15.098731 [PREPRINT] PMC7499305 32943628

[emmm202013105-bib-0100] Sharma A (2020) Chloroquine paradox may cause more damage than help fight COVID‐19. Microbes Infect 22: 154–156 32305500 10.1016/j.micinf.2020.04.004 PMC7162740

[emmm202013105-bib-0101] Sheahan TP , Sims AC , Graham RL , Menachery VD , Gralinski LE , Case JB , Leist SR , Pyrc K , Feng JY , Trantcheva I *et al* (2017) Broad‐spectrum antiviral GS‐5734 inhibits both epidemic and zoonotic coronaviruses. Sci Transl Med 9: eaal3653 28659436 10.1126/scitranslmed.aal3653 PMC5567817

[emmm202013105-bib-0102] Sheahan TP , Sims AC , Leist SR , Schafer A , Won J , Brown AJ , Montgomery SA , Hogg A , Babusis D , Clarke MO *et al* (2020) Comparative therapeutic efficacy of remdesivir and combination lopinavir, ritonavir, and interferon beta against MERS‐CoV. Nat Commun 11: 222 31924756 10.1038/s41467-019-13940-6 PMC6954302

[emmm202013105-bib-0103] Shirato K , Kawase M , Matsuyama S (2018) Wild‐type human coronaviruses prefer cell‐surface TMPRSS2 to endosomal cathepsins for cell entry. Virology 517: 9–15 29217279 10.1016/j.virol.2017.11.012 PMC7112029

[emmm202013105-bib-0104] Sieczkarski SB , Whittaker GR (2002) Dissecting virus entry via endocytosis. J Gen Virol 83: 1535–1545 12075072 10.1099/0022-1317-83-7-1535

[emmm202013105-bib-0105] Sigrist CJ , Bridge A , Le Mercier P (2020) A potential role for integrins in host cell entry by SARS‐CoV‐2. Antiviral Res 177: 104759 32130973 10.1016/j.antiviral.2020.104759 PMC7114098

[emmm202013105-bib-0106] Spinner CD , Gottlieb RL , Criner GJ , Arribas López JR , Cattelan AM , Soriano Viladomiu A , Ogbuagu O , Malhotra P , Mullane KM , Castagna A *et al* (2020) Effect of remdesivir vs standard care on clinical status at 11 days in patients with moderate COVID‐19: a randomized clinical trial. JAMA 324: 1048 32821939 10.1001/jama.2020.16349 PMC7442954

[emmm202013105-bib-0107] Streeter DG , Witkowski JT , Khare GP , Sidwell RW , Bauer RJ , Robins RK , Simon LN (1973) Mechanism of action of 1‐ ‐D‐ribofuranosyl‐1,2,4‐triazole‐3‐carboxamide (Virazole), a new broad‐spectrum antiviral agent. Proc Natl Acad Sci USA 70: 1174–1178 4197928 10.1073/pnas.70.4.1174 PMC433451

[emmm202013105-bib-0108] Sungnak W , Huang N , Bécavin C , Berg M , Queen R , Litvinukova M , Talavera‐López C , Maatz H , Reichart D , Sampaziotis F *et al* (2020) SARS‐CoV‐2 entry factors are highly expressed in nasal epithelial cells together with innate immune genes. Nat Med 26: 681–687 32327758 10.1038/s41591-020-0868-6 PMC8637938

[emmm202013105-bib-0109] Tchesnokov EP , Feng JY , Porter DP , Gotte M (2019) Mechanism of inhibition of ebola virus RNA‐dependent RNA Polymerase by Remdesivir. Viruses 11: 326 10.3390/v11040326 PMC6520719 30987343

[emmm202013105-bib-0110] To KK , Tsang OT , Leung WS , Tam AR , Wu TC , Lung DC , Yip CC , Cai JP , Chan JM , Chik TS *et al* (2020) Temporal profiles of viral load in posterior oropharyngeal saliva samples and serum antibody responses during infection by SARS‐CoV‐2: an observational cohort study. Lancet Infect Dis 20: 565–574 32213337 10.1016/S1473-3099(20)30196-1 PMC7158907

[emmm202013105-bib-0111] van der Lugt J , Lange J , Avihingsanon A , Ananworanich J , Sealoo S , Burger D , Gorowara M , Phanuphak P , Ruxrungtham K (2009) Plasma concentrations of generic lopinavir/ritonavir in HIV type‐1‐infected individuals. Antivir Ther 14: 1001–1004 19918104 10.3851/IMP1410

[emmm202013105-bib-0112] Vanderlinden E , Vrancken B , Van Houdt J , Rajwanshi VK , Gillemot S , Andrei G , Lemey P , Naesens L (2016) Distinct effects of T‐705 (Favipiravir) and ribavirin on influenza virus replication and viral RNA synthesis. Antimicrob Agents Chemother 60: 6679–6691 27572398 10.1128/AAC.01156-16 PMC5075073

[emmm202013105-bib-0113] Varghese FS , Thaa B , Amrun SN , Simarmata D , Rausalu K , Nyman TA , Merits A , McInerney GM , Ng LFP , Ahola T (2016) The antiviral alkaloid berberine reduces chikungunya virus‐induced mitogen‐activated protein kinase signaling. J Virol 90: 9743–9757 27535052 10.1128/JVI.01382-16 PMC5068526

[emmm202013105-bib-0114] Villalaín J (2010) Membranotropic effects of arbidol, a broad anti‐viral molecule, on phospholipid model membranes. J Phys Chem B 114: 8544–8554 20527735 10.1021/jp102619w

[emmm202013105-bib-0115] Vincent MJ , Bergeron E , Benjannet S , Erickson BR , Rollin PE , Ksiazek TG , Seidah NG , Nichol ST (2005) Chloroquine is a potent inhibitor of SARS coronavirus infection and spread. Virol J 2: 69 16115318 10.1186/1743-422X-2-69 PMC1232869

[emmm202013105-bib-0116] Walls AC , Park YJ , Tortorici MA , Wall A , McGuire AT , Veesler D (2020) Structure, function, and antigenicity of the SARS‐CoV‐2 spike glycoprotein. Cell 181: 281–292 32155444 10.1016/j.cell.2020.02.058 PMC7102599

[emmm202013105-bib-0117] Wang H , Yang P , Liu K , Guo F , Zhang Y , Zhang G , Jiang C (2008) SARS coronavirus entry into host cells through a novel clathrin‐ and caveolae‐independent endocytic pathway. Cell Res 18: 290–301 18227861 10.1038/cr.2008.15 PMC7091891

[emmm202013105-bib-0118] Wang M , Cao R , Zhang L , Yang X , Liu J , Xu M , Shi Z , Hu Z , Zhong W , Xiao G (2020a) Remdesivir and chloroquine effectively inhibit the recently emerged novel coronavirus (2019‐nCoV) in vitro. Cell Res 30: 269–271 32020029 10.1038/s41422-020-0282-0 PMC7054408

[emmm202013105-bib-0119] Wang X , Cao R , Zhang H , Liu J , Xu M , Hu H , Li Y , Zhao L , Li W , Sun X (2020b) The anti‐influenza virus drug, arbidol is an efficient inhibitor of SARS‐CoV‐2 in vitro. Cell Discov 6: 28 32373347 10.1038/s41421-020-0169-8 PMC7195821

[emmm202013105-bib-0120] Wang Y , Zhang D , Du G , Du R , Zhao J , Jin Y , Fu S , Gao L , Cheng Z , Lu Q *et al* (2020c) Remdesivir in adults with severe COVID‐19: a randomised, double‐blind, placebo‐controlled, multicentre trial. Lancet 395: 1569–1578 32423584 10.1016/S0140-6736(20)31022-9 PMC7190303

[emmm202013105-bib-0121] Warren TK , Jordan R , Lo MK , Ray AS , Mackman RL , Soloveva V , Siegel D , Perron M , Bannister R , Hui HC *et al* (2016) Therapeutic efficacy of the small molecule GS‐5734 against Ebola virus in rhesus monkeys. Nature 531: 381–385 26934220 10.1038/nature17180 PMC5551389

[emmm202013105-bib-0122] Wellems TE , Plowe CV (2001) Chloroquine‐resistant malaria. J Infect Dis 184: 770–776 11517439 10.1086/322858

[emmm202013105-bib-0123] Williamson BN , Feldmann F , Schwarz B , Meade‐White K , Porter DP , Schulz J , van Doremalen N , Leighton I , Yinda CK , Pérez‐Pérez L *et al* (2020) Clinical benefit of remdesivir in rhesus macaques infected with SARS‐CoV‐2. Nature 585: 273–276 32516797 10.1038/s41586-020-2423-5 PMC7486271

[emmm202013105-bib-0124] Wölfel R , Corman VM , Guggemos W , Seilmaier M , Zange S , Müller MA , Niemeyer D , Jones TC , Vollmar P , Rothe C *et al* (2020) Virological assessment of hospitalized patients with COVID‐2019. Nature 581: 465–469 32235945 10.1038/s41586-020-2196-x

[emmm202013105-bib-0125] Wollheim FA , Hanson A , Laurell CB (1978) Chloroquine treatment in rheumatoid arthritis. Correlation of clinical response to plasma protein changes and chloroquine levels. Scand J Rheumatol 7: 171–176 364636 10.3109/03009747809095649

[emmm202013105-bib-0126] Wrapp D , Wang N , Corbett KS , Goldsmith JA , Hsieh CL , Abiona O , Graham BS , McLellan JS (2020) Cryo‐EM structure of the 2019‐nCoV spike in the prefusion conformation. Science 367: 1260–1263 32075877 10.1126/science.abb2507 PMC7164637

[emmm202013105-bib-0127] Wray SK , Gilbert BE , Noall MW , Knight V (1985) Mode of action of ribavirin: effect of nucleotide pool alterations on influenza virus ribonucleoprotein synthesis. Antiviral Res 5: 29–37 3985606 10.1016/0166-3542(85)90012-9

[emmm202013105-bib-0128] Xia S , Lan Q , Su S , Wang X , Xu W , Liu Z , Zhu Y , Wang Q , Lu L , Jiang S (2020a) The role of furin cleavage site in SARS‐CoV‐2 spike protein‐mediated membrane fusion in the presence or absence of trypsin. Signal Transduct Target Ther 5: 92 32532959 10.1038/s41392-020-0184-0 PMC7289711

[emmm202013105-bib-0129] Xia S , Zhu Y , Liu M , Lan Q , Xu W , Wu Y , Ying T , Liu S , Shi Z , Jiang S *et al* (2020b) Fusion mechanism of 2019‐nCoV and fusion inhibitors targeting HR1 domain in spike protein. Cell Mol Immunol 17: 765–767 32047258 10.1038/s41423-020-0374-2 PMC7075278

[emmm202013105-bib-0130] Yao X , Ye F , Zhang M , Cui C , Huang B , Niu P , Liu X , Zhao L , Dong E , Song C *et al* (2020) In Vitro Antiviral Activity and Projection of Optimized Dosing Design of Hydroxychloroquine for the Treatment of Severe Acute Respiratory Syndrome Coronavirus 2 (SARS‐CoV‐2). Clin Infect Dis 71(15): 732–739 32150618 10.1093/cid/ciaa237 PMC7108130

[emmm202013105-bib-0131] Yates MK , Seley‐Radtke KL (2019) The evolution of antiviral nucleoside analogues: A review for chemists and non‐chemists. Part II: Complex modifications to the nucleoside scaffold. Antiviral Res 162: 5–21 30529089 10.1016/j.antiviral.2018.11.016 PMC6349489

[emmm202013105-bib-0132] Zhang C , Shi L , Wang FS (2020a) Liver injury in COVID‐19: management and challenges. Lancet Gastroenterol Hepatol 5: 428–430 32145190 10.1016/S2468-1253(20)30057-1 PMC7129165

[emmm202013105-bib-0133] Zhang L , Lin D , Sun X , Curth U , Drosten C , Sauerhering L , Becker S , Rox K , Hilgenfeld R (2020b) Crystal structure of SARS‐CoV‐2 main protease provides a basis for design of improved α‐ketoamide inhibitors. Science 368: 409–412 32198291 10.1126/science.abb3405 PMC7164518

[emmm202013105-bib-0134] Zhou R , Li F , Chen F , Liu H , Zheng J , Lei C , Wu X (2020) Viral dynamics in asymptomatic patients with COVID‐19. Int J Infect Dis 96: 288–290 32437933 10.1016/j.ijid.2020.05.030 PMC7211726

[emmm202013105-bib-0135] Zhu N , Zhang D , Wang W , Li X , Yang B , Song J , Zhao X , Huang B , Shi W , Lu R *et al* (2020) A novel coronavirus from patients with pneumonia in China, 2019. N Engl J Med 382: 727–733 31978945 10.1056/NEJMoa2001017 PMC7092803

